# Coupled SDE-ODE Modeling of Tumor-Immune Dynamics to Infer Biomarker Release

**DOI:** 10.1007/s11538-026-01665-9

**Published:** 2026-06-23

**Authors:** Pujan Shrestha, Yijia Fan, Jason T. George

**Affiliations:** 1 Department of Biomedical Engineering, Texas A&M University, College Station, 77843 TX USA; 2 Translational Medical Sciences, Texas A&M Health Science Center, Houston, 77005 TX USA; 3 Center for Theoretical Biological Physics, Rice University, Street, Houston, 77005 Houston USA; 4 Hematopoietic Biology and Malignancy, MD Anderson Cancer Center, Houston, 77030 TX USA

**Keywords:** Mathematical Oncology, Stochastic Differential Equations, Ordinary Differential Equations, Biomarker Modeling

## Abstract

Tumor–immune interactions are central to cancer progression and treatment response, driving cell death through immune-mediated killing and resource-limited competition. In early-stage disease or following effective treatment, cancer populations are often small and difficult to observe directly. Disease monitoring therefore relies on the detection of biomarkers such as circulating tumor DNA (ctDNA) as noisy proxies to cancer size. Existing approaches lack robust frameworks to infer tumor burden from these signals when populations approach detection thresholds. We present a coupled deterministic–stochastic framework that links tumor–immune dynamics to biomarker release. A two prey, one predator Lotka–Volterra model captures interactions between immune cells and competing tumor subpopulations under shared resource constraints. Biomarker production is modeled using stochastic differential equations driven by tumor cell death arising from immune-mediated apoptosis and intratumor competition–induced necrosis. We incorporate both square-root (CIR-type) noise, representing count-limited fluctuations near the detection limit, and multiplicative (geometric-type) noise, representing proportional variability at higher concentrations. Within this framework, we derive analytical expressions for biomarker trajectories and first-passage statistics, including mean detection times under minimal detection thresholds. Our results demonstrate how tumor heterogeneity, immune pressure, and stochastic variance structure jointly shape biomarker emergence, persistence, and detectability. Together, this work provides a mechanistically interpretable and mathematically tractable foundation for inferring tumor burden from noisy biomarker signals across clinically relevant concentration regimes.

**Supplementary Information:**

The online version contains supplementary material available at 10.1007/s11538-026-01665-9.

## Introduction

The adaptive immune system plays an important role in shaping cancer development, and tumor-immune interactions lead to one of three possible clinical outcomes: tumor escape, tumor elimination, or a period of sustained equilibrium (Dunn et al. 2002; Schreiber et al. 2011). In the elimination phase, CD8+ cytotoxic T lymphocytes (CTL) and natural killer (NK) cells recognize and destroy tumor cells through tumor-associated antigens (TAA) presented on the tumor major histocompatibility complex (MHC) (Dunn et al. 2002). Cancer cells may enter into a state of equilibrium with an active immune system if they can avoid complete destruction. Such states are reflected by interesting dynamical properties that give rise to potentially large waiting times (George and Levine 2020; Wang et al. 2019). Over time, malignant cells can become more immune evasive through a variety of mechanisms, including down-regulation of antigen presentation machinery, up-regulation of immune checkpoint molecules (e.g. PD-L1), loss of recognized tumor-associated antigens, and secretion of immunosuppressive cytokines (Schreiber et al. 2011; Ribas 2015; George and Levine 2018, 2021; Kayhanian et al. 2024; George and Levine 2023).

Despite growing insights into tumor–immune interactions, a current major limitation to understanding these details more precisely remains in the ability to accurately infer tumor burden from circulating biomarkers, particularly when disease levels are low (Alizadeh et al. 2015). One existing and clinically relevant example is circulating tumor DNA (ctDNA) released during apoptotic and necrotic cell death (Li et al. 2025; Bettegowda et al. 2014). However, its interpretation is complicated by stochastic shedding dynamics, contributions from heterogeneous tumor subpopulations, and noise introduced by sampling and clearance processes (Diehl et al. 2008; Mouliere et al. 2018; Newman et al. 2014). These factors become especially limiting near detection thresholds, where signal-to-noise ratios deteriorate and conventional inference methods often fail to recover underlying tumor dynamics (Dawson et al. 2013; Kurtz et al. 2021). A key unmet need is a principled framework that links observed biomarker fluctuations to population-level tumor behavior, particularly under conditions of uncertainty. Analogous challenges arise in chronic infections such as HIV and hepatitis C, where low-level residual disease may persist below detection yet remain biologically relevant (Palmer et al. 2008; Shoukry et al. 2004).

To address these challenges, we develop a hybrid mathematical framework that couples deterministic tumor–immune dynamics with an underlying stochastic model of biomarker release. We introduce an ordinary differential equation (ODE) model of tumor–immune interaction based on a predator–prey (immune-tumor) structure (Kuznetsov et al. 1994; Kirschner and Panetta 1998; Kareva and Berezovskaya 2015; Kareva et al. 2010; Aguadé-Gorgorió et al. 2024; Wilkie 2013; Yang et al. 2025). The structure of our model draws from ecological predator–prey theory, where multiple prey species interacting with a shared predator have long provided insight into coexistence and competition. In these systems, predators can couple the dynamics of otherwise independent prey species, producing indirect interactions and modifying coexistence conditions (Chesson 2000; Abrams and Matsuda 1996; Yang et al. 2025). Mathematical analyses of multi-species Lotka-Volterra (LV) systems establish general criteria for stability and persistence in such networks (Hofbauer and Sigmund 1998; Takeuchi 1996), and recent work has examined permanence in multi-prey predator–prey systems directly (Schreiber and Patel 2015). We adopt this ecological perspective by treating two tumor subpopulations as prey populations regulated by a shared immune predator, allowing us to capture competitive and cooperative interactions mediated through immune pressure. Cancer cells are divided into immune-targeted and immune-evasive subpopulations, allowing the model to capture selection under variable fitness costs incurred under immune pressure. Immune-targeted cells are eliminated through immune-mediated apoptosis, represented by Lotka–Volterra-type interaction terms, while logistic growth constraints lead to necrotic death under high tumor burden (Gatenby and Gawlinski 1996; Kanduc et al. 2002).

We note that classical LV systems can exhibit oscillatory behavior that is not always reflected in measured tumor volumes. In this work, the LV structure is not used to replicate empirical tumor growth curves, but rather to encode mechanistic interactions—competition between tumor phenotypes and immune-mediated predation—within a mathematically tractable framework. In our setting, logistic growth constraints and immune-induced death terms limit the emergence of sustained cyclic dynamics. Accordingly, we use the LV formulation as a structured backbone for incorporating biologically relevant regulatory mechanisms.

Building on this foundation, we introduce a stochastic differential equation (SDE) that links tumor cell death, via both apoptosis and necrosis, to the generation of ctDNA and other measurable biomarkers (Diehl et al. 2008; Stejskal et al. 2023; Ribba et al. 2023; Barlebo Ahlborn and Østrup 2019). This stochastic component captures the intrinsic variability in biomarker shedding, clearance, and measurement noise. Apoptotic and necrotic processes contribute to ctDNA through distinct mechanisms (for example, immune-mediated fragmentation versus passive lysis), allowing the model to distinguish their molecular signatures. Importantly, empirical studies show that ctDNA variability operates in two characteristic regimes that motivate our modeling choices: 1) near the detection limit, where measurement noise is dominated by Poisson sampling, reflecting the fundamental uncertainty that arises when only a few DNA fragments are present (Tellinghuisen 2020; Ye et al. 2025), and 2) at moderate and higher concentrations, where technical and biological fluctuations behave multiplicatively, with noise increasing approximately in proportion to the amount of DNA measured (Henriksen et al. 2022; Markovets et al. 2025). These observations guide the stochastic structure we adopt in later sections, allowing the model to distinguish regimes of low-count Poisson behavior from those where proportional, multiplicative variability is more appropriate.

By modeling heterogeneity in immune targeting under shared resource constraints, we capture a range of escape and coexistence outcomes that depend on mutual altruism and egoism between the subpopulations under differential immune targeting. We then extend this system using stochastic differential equations to track the noisy evolution of biomarker signals, including those arising from apoptosis and necrosis. This formulation allows us to analytically characterize when and how such signals become detectable, and to identify key parameters that influence early detection. Together, our findings provide a theoretical foundation for interpreting noisy biomarker data in the context of immune surveillance, and suggest design principles for improving detection of minimal residual disease.

## Methods and Materials

### Model Development

**Fig. 1:**
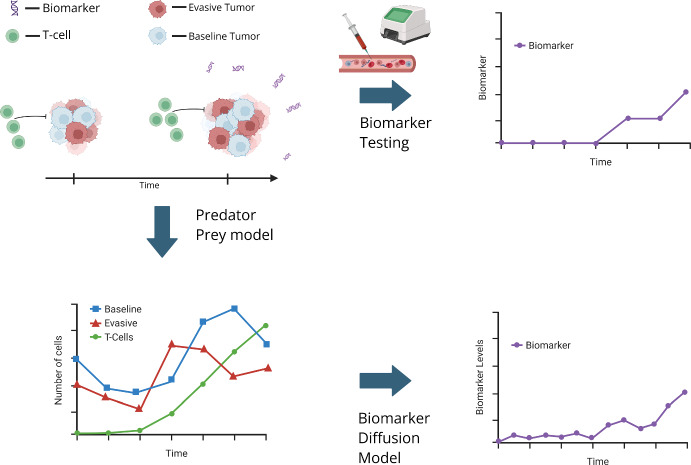
Conceptual schematic for the ODE-SDE model in biomarker trajectories. Tumor populations evolve under immune pressure, giving rise to heterogeneous dynamics between an immune-sensitive ‘baseline’ subpopulation and immune-evasive cells. These interactions, along with immune-mediated killing, drive biomarker release into circulation through apoptotic and necrotic cell death. While the underlying population dynamics (bottom left) remain unobservable, they influence measurable biomarker levels (top right), which exhibit stochastic fluctuations due to variability in shedding and clearance. Coupling deterministic tumor-immune dynamics with stochastic biomarker shedding provides a framework for inferring hidden cancer population states from noisy biomarker data

We present a generalized framework for modeling interactions between a heterogeneous population of cancer cells and the immune system, combining deterministic tumor growth and immune response dynamics with stochastic variability in biomarker release (Fig. 1). We assume that the tumor subpopulations and T cells behave according to a one-predator, two-prey Lotka-Volterra model. Furthermore, we assume that, in addition to T cell elimination pressure, the two tumor subpopulations are also competing with each other under a shared carrying capacity with parameters denoting the degree of altruism vs egoism. Such social behavior as altruism has been previously documented in breast cancers and is a subject of active research (Masroni et al. 2023). In previous work, the authors constructed a mathematical model where immunomodulation would impact the per-cell killing rate of the birth-death process representing tumor population (Shrestha et al. 2025). Under this context, this immunomodulation can be represented as changes in the T cell interaction and expansion terms.

#### Tumor Immune Dynamics

Let *B*, *E*, and *I* represent the baseline tumor, evasive tumor, and T cell compartments, respectively. The three compartments are linked via a system of ODEs, given by:

1 $$\begin{aligned} dE&= r E \left( 1 - \frac{E + p B}{K}\right) - \alpha E I, \nonumber \\ dB&= \gamma B \left( 1 - \frac{q E + B}{K}\right) - \beta B I, \nonumber \\ dI&= a E I + b B I - \delta I \end{aligned}$$

with $E(0), B(0) , I(0) > 0$, and $r, \gamma , \alpha , \beta , a,b,\delta > 0$ as the model parameters with $\alpha < \beta$. We consider a finite time horizon, $0 \le t \le T$ for some $T \ge 0$. The parameter definitions are provided in Table 1.

**Table 1:** Model parameters and their descriptions

Parameter(s)	Description
*K*	Carrying capacity for the tumors
$r, \gamma$	Tumor growth rates
$\alpha , \beta$	Tumor-immune interaction tumor death rates, with $\alpha < \beta$
*a*, *b*	T cell growth rates resulting from tumor-immune interaction
$\delta$	T cell death rate
*p*, *q*	Scaling parameters for competition between cancer populations under shared carrying capacity

We define the competitive parameters *p* and *q* to represent the relative level of altruism/egoism of each subpopulation, as has been modeled previously (Vibishan et al. 2025). In this interpretation, egoistic and altruistic behaviors could be seen as regimes for the parameters. For example, when $q > 1$, the baseline tumor places more weight on the evasive tumor volume in modulating its own growth rate. On the other hand, $0< q < 1$ gives the opposite behavior. Finally, we remark that the high number of parameters presents a challenge for future data fitting and modeling. To mitigate this, we perform time and population scaling relative to a characteristic immune compartment size $I_0$ to obtain an alternate dimensionless version of the model with a reduced number of (six) parameters, given by (1). For more details, refer to Sec S1 of SI.

#### Biomarker Dynamics

The detailed physiological processes underlying biomarker kinetics are complex and often involve multiple independent contributions to overall dynamics. For foundational understanding, we motivate our framework by considering the example of ctDNA-a biomarker that has previously been used as a specific indicator of tumor burden and is often most informative when measured in the regime of low tumor cell populations, near the detection limit. Moreover, ctDNA release into the bloodstream occurs through apoptosis or necrosis, with the release rate being approximately proportional to tumor size (Stejskal et al. 2023; Diehl et al. 2008; Mouliere et al. 2011; Bettegowda et al. 2014). Similarly, (primary renal and hepatic) clearance mechanisms follow first-order kinetics, implying that the rate of biomarker decay is proportional to its current concentration (Ribba et al. 2023; Li et al. 2025).

To motivate the stochastic component, we note that ctDNA quantification operates in two empirically supported noise regimes. Near the detection limit, variability is dominated by Poisson sampling: precision in dPCR/qPCR fundamentally scales with the number of template molecules (Tellinghuisen 2020), and when only a handful of variant fragments are present, the coefficient of variation becomes large (Ye et al. 2025). This supports modeling ultra–low counts using a square-root diffusion (CIR/Feller-type) with diffusion term proportional to $\sqrt{C}$:

2 $$\begin{aligned} dC&= \big ( f(E,B,I) - \eta C \big ) dt + \sigma \sqrt{C} \, dW(t) \end{aligned}$$

where the diffusion term scales as $\sqrt{C}$, consistent with Poisson-driven fluctuations in low-molecule-count conditions. In particular, $\sigma \sqrt{C}\,dW(t)$ represents the count-limited finite-molecule regime where Poisson noise from sampling rare fragments imposes a fundamental precision limit in PCR/dPCR quantification (Tellinghuisen 2020; Quan et al. 2018).

At moderate and higher concentrations, technical and biological variability instead behave multiplicatively: noise increases roughly in proportion to DNA input (Henriksen et al. 2022), coefficient of variation stabilizes across clinically relevant allele fractions (Ye et al. 2025), and ctDNA levels show intrinsic proportional fluctuations even in the absence of treatment (Markovets et al. 2025). In this regime, stochasticity is approximated by noise acting on $\log C$, yielding diffusion proportional to *C*. As such, and in line with prior stochastic modeling frameworks of ctDNA dynamics (Avanzini et al. 2020), we also represent *C*(*t*) by the SDE:

3 $$\begin{aligned} dC&= \big ( f(E,B,I) - \eta C \big ) dt + \sigma C \, dW(t) \end{aligned}$$

where the diffusion term is proportional to *C*, producing geometric-type variability and an approximately constant coefficient of variation across clinically relevant ctDNA levels. Here, $\sigma C\,dW(t)$ is intended as an effective model once additional variability sources scale with DNA input and Poisson-only sampling CV is no longer the dominant contribution.

Thus, depending on the regime of interest, we use families of SDEs to capture both deterministic influences from tumor-immune interactions and inherent biological variability in biomarker levels and their consequent detection. In both of these noise regimes, $C(0) > 0$, *W*(*t*) is standard Brownian motion and $\sigma , \eta$ are the volatility and the decay rates, respectively. We assume that apoptotic events occur via the interactions between tumor and T cells. As such, we consider such interactions in the appropriate tumor subpopulation as well as the joint release for the shedding mechanism. Similarly, the necrotic release is tracked by the “over-crowding” term that arises from the shared carrying capacity. Using the longitudinal population dynamics from the ODE model, we model the apoptotic and necrotic release for each tumor subpopulation. Consequently, the total biomarker release summed over evasive/baseline and apoptotic/necrotic contributions becomes a linear combination of these interaction terms. In Table 2, we list different functional forms of interest for *f*. Note that the model assumes a universal decay and volatility factor common to all release mechanisms. For this paper, we assume that *W*(*t*) in the SDE for one release mechanism is independent and identically distributed to the Brownian noise of the other SDEs.

**Table 2:** Biomarker trajectory expressions for different tumor and cell death dynamics

Biomarker	Expression *f*(*E*, *B*, *I*)
Evasive Tumor	$r E \left( \frac{E + p B}{K} \right) + \alpha E I$
Baseline Tumor	$\gamma B \left( \frac{q E + B}{K} \right) + \beta B I$
Apoptotic Cells	$\alpha E I + \beta B I$
Necrotic Cells	$\gamma B \left( \frac{q E + B}{K} \right) + r E \left( \frac{E + p B}{K} \right)$

### Numerical Simulations

The ODE was solved via the numpy and scipy library on Python. For the stochastic differential equations, the numerical solutions were obtained via Euler-Maruyama scheme and path solution simulations were generated via Monte Carlo simulations. Github code for the model is available at https://github.com/TAMUGeorgeGroup/ODE_SDE_Model.

## Results

Below, we summarize our key findings. We refer the reader to the appendices for complete mathematical details.

### Dynamical Behavior of the Tumor-Immune System

#### Theorem 1

(Equilibrium states and Local stability) The equilibrium states and their existence and stability conditions for the system of ODEs given by (1) are given in Table 3.

**Table 3:** Parameter dependence on existence and local stability of Equilibrium states

	Equilibrium States	Conditions for Existence	Conditions for Stability
(*i*)	(0, 0, 0)	Exists	Unstable
(*ii*)	(*K*, 0, 0)	Exists	$q > 1$ and $K < \frac{\delta }{a}$
(*iii*)	(0, *K*, 0)	Exists	$p > 1$ and $K < \frac{\delta }{b}$
(*iv*)	$\left( \dfrac{K(1-p)}{1-pq}, \dfrac{K(1-q)}{1-pq}, 0 \right)$	$pq \ne 1$ and ($p,q > 1$ or $p,q < 1$)	$\delta > \dfrac{K}{1-pq} \left( a(1-p) + b(1-q)\right)$
(*v*)	$\left( \dfrac{\delta }{a}, 0, \dfrac{r}{\alpha } \left( 1 - \dfrac{\delta }{a K}\right) \right)$	$K > \dfrac{\delta }{a}$	$q > q_0$
(*vi*)	$\left( 0 , \dfrac{\delta }{b}, \dfrac{\gamma }{\beta } \left( 1 - \dfrac{\delta }{b K}\right) \right)$	$K > \dfrac{\delta }{b}$	$p > p_0$
(*vii*)	$(\hat{E}, \hat{B}, \hat{I})^*$	($p,q > p_0, q_0$ or $p,q< p_0, q_0$) and $p,q < p_{max}, q_{max}$*	$\bigstar$

Here $p_0$, $q_0$, $p_{max}$, $q_{max}$ are constants dependent on the parameter values. Furthermore, for $\bigstar$, we apply the Routh-Hurwitz stability criterion (Anagnost and Desoer 1991) on the characteristic equation for the interior state to obtain stability conditions.

#### Proof

Section A.2 of the Appendix. $\square$

Restriction of the solution to the positive octant allows us to find parametric restrictions for existence of the equilibrium points. Theorem 1 provides an analytical perspective on the local stability of these equilibrium points. Note that the Lotka-Volterra system in Eq. (1) is nonlinear. For the local stability results, we performed linearization in a neighborhood of each equilibrium state, and as such, the resulting stability conclusions apply only locally. These linearization-based results should therefore not be interpreted as describing the global behavior of the system, which we analyze separately using Lyapunov methods. We describe these states below, which are summarized in Table 3.

The state where all compartments vanish (Table 3(*i*)) is unstable for all parametric combinations. The instability of the tumor-free equilibrium in the deterministic ODE formulation is a mathematical artifact of the large-population limit rather than a biological statement about the impossibility of tumor extinction. In contrast, a stochastic birth–death process naturally allows the tumor population to hit zero with positive probability (Fan and George 2025). Demographic fluctuations drive some realizations to extinction, consistent with both clinical spontaneous regression and experimental observations in mouse models. We therefore emphasize that the instability of the origin in the deterministic model should not be interpreted as a biological impossibility of immune-mediated tumor clearance; rather, it reflects a limitation of the ODE approximation on continuous state space.

The tumor competition parameters play a key role in the existence and stability of the remaining equilibrium states. When looking at evasive homogeneous escape state in Table 3(*ii*), the immune evasive cancer population grows to carrying capacity with mutual extinction of both baseline cancer and adaptive immune populations whenever a) the baseline tumor exhibits altruism ($q>1$) and b) the ratio of the T cell death rate to the T cell growth rate via the interactions between the T cells and the evasive tumor exceeds the carrying capacity. Interchanging the roles of parameters specific to the baseline and evasive cancer subpopulation gives rise to a solitary baseline cancer population growing to carrying capacity (Table 3(*iii*)).

In heterogeneous tumor escape state (Table 3(iv)), both the baseline and evasive equilibrium states themselves are scaled by the competition parameters, while existence requires both tumor populations would have to be simultaneously either egoistic ($p< 1, \, q < 1$) or altruistic ($p> 1, \, q > 1$) for such a condition to even exist. This result aligns well with the game-theoretic intuition that populations with mixed strategies would lead to the dominance of the egoistic strategist.

The homogeneous tumor-immune coexistence states (Table 3(*v*), (*vi*)), wherein one of the tumor subpopulation strikes a balance with the immune system while the other tumor population faces extinction, provide additional insight into the interplay of the competitive forces of the tumor subtypes. We obtain a lower bound on the competition parameters, $p_0$ or $q_0$, required for stability in the cases where only one cancer subpopulation survives. In particular, the dying subpopulation must exhibit altruism in excess of a lower bound on the population’s respective competition parameter. These lower bounds can be obtained from Eqs. A9 and A10 in the appendix and are given by

4 $$\begin{aligned} p_0 \triangleq 1 + \left( \frac{bK}{\delta } - 1 \right) \left( 1 - \frac{\gamma \alpha }{r \beta }\right) , \nonumber \\ q_0 \triangleq 1 +\left( \frac{aK}{\delta } - 1 \right) \left( 1 - \frac{r \beta }{\gamma \alpha }\right) . \end{aligned}$$

These bounds are modulated by the relative competitive advantage between the cancer subpopulations and the relative free space left under shared carrying capacity. Moreover, they act independently for the homogeneous tumor-immune coexistence state. However, we find a mutual coaction is required for the interior equilibrium state given by Table 3(*vii*). This is perhaps the most interesting state, wherein both cancer subpopulations co-exist with the immune compartment under a shared carrying capacity leading to a heterogeneous immune-mediated coexistence state. Existence requires both tumor populations to be simultaneously either more egoistic or more altruistic than the lower bounds given in (4). More concretely, if $p> p_0,\, q > q_0$ and the evasive cancer subpopulation enjoys a competitive advantage ($\beta> \alpha , r > \gamma$), then the evasive tumor is more likely to fully occupy the carrying capacity leading to a homogeneous coexistence state or immune escape state. We can use the relation $r \beta > \gamma \alpha$ to obtain $p_0> 1 > q_0$. Thus, the evasive tumor has to be altruistic ($p> p_0 > 1$) while the baseline tumor is allowed a little bit of egoism ($q \in (q_0 , 1) \cup [1 , \infty )$). The interior state therefore requires a balance between the immune forces and the competitive forces for existence and stability. Note that the tumor-immune coexistence state when considering low tumor populations overlaps with empirically observed immune-mediated dormancy (Wang et al. 2019).

Intriguingly, $pq \ne 1$ emerges as a critical condition for immune-mediated dormant states for which the tumor subpopulations strike a balance with the immune compartment resulting in a net zero growth rate. Indeed, for the interior equilibrium with non-zero values, $p = q = 1$ would imply that either, at equilibrium, $I = 0$ contradicting the non zero assumption or there exists a constant $k_0$ such that $\gamma \alpha = k_0 r \beta$. In the latter case, $dE = k_0 dS$ which would mean that the evasive population is nothing but a scaled version of the baseline one. Under this very restrictive condition on the competitive rates, the two cancer subpopulations match exactly in their dynamics and as such, can be viewed as a single (homogeneous) population.

#### Global Stability of the Tumor-Immune System

The system of ODEs given in (1) is nonlinear and as such, stability analysis via the linearized system is restricted to describing the behavior of trajectories close to equilibrium. We investigate global stability for the interior equilibrium state via Lyapunov’s second method of stability.

##### Theorem 2

(Lyapunov Stability) Consider the following functional *L* given by

$$\begin{aligned} L(E, B ,I)&= p \left( E - \hat{E} - \hat{E} \ln {\frac{E}{ \hat{E}}} \right) + \frac{b \alpha p}{ a \beta } \left( B - \hat{B} - \hat{B} \ln {\frac{B}{ \hat{B} }} \right) \\&+ \frac{\alpha p}{a} \left( I - \hat{I} - \hat{I} \ln {\frac{I}{ \hat{I} }} \right) , \end{aligned}$$

where $\hat{E}, \hat{B}, \hat{I}$ are the interior state values. Then *L* is positive definite and zero only at the interior equilibrium. Furthermore, $\frac{dL}{dt} < 0$ and consequently, *L* is a Lyapunov functional when either of the following conditions hold:

- $$E > \hat{E} $$ and $$ B > \hat{B}, $$
- $$E < \hat{E} $$ and $$ B < \hat{B}. $$

##### Proof

Section A.3 of Appendix. $\square$

The functional form of *L* was chosen to measure deviations from the equilibrium while allowing for easier tractability with the time derivative operator. We find that trajectories where the tumor population size is either jointly above or below the interior equilibrium size will stably converge to the equilibrium globally asymptotically. However, fluctuations in the trajectories when the above conditions are not satisfied might lead to possible extinction of at least one subcompartment.

### Dynamical Behavior of the Biomarker Compartment

So far we have carefully analyzed the deterministic arm of the model which is meant to track tumor-immune interactions. To get a better understanding of how the biomarker release reflects the longitudinal tumor-immune dynamics, we consider the stochastic differential equation given by (3) and assume that the drift terms for the biomarker release mechanisms are driven by apoptotic and necrotic events in the tumor-immune microenvironment: apoptotic via tumor-immune killing, necrotic via carrying capacity-induced cell death. Let $c^*$ be the minimal biomarker size required for the tumor to be detected. The model dynamics from the competitive Lotka-Volterra system serve as an input to the time-dependent drift for the biomarker model. We remark that the general form of the drift term enables the modeling of the biomarker trajectory in multiple biological contexts depending on the nature of the signal source. For foundational understanding, we normalize the biomarker level by $c^*$. Under this assumption, we consider the possible forms and rationale for the drift term *f* (Table 4). Note that this is the the same release assumptions, albeit normalized as in Table 2: apoptotic via tumor-immune interaction and necrotic via carrying capacity related deaths.

**Table 4:** Form of *f* for normalized evasive and baseline tumors

Evasive	$\frac{1}{c^*}\left( \alpha E I + r E ( \frac{E + p B}{K}) \right)$
Baseline	$\frac{1}{c^*}\left( \beta B I + \gamma B ( \frac{q E + B}{K})\right)$
Apoptotic	$\frac{1}{c^*}\left( \alpha E I + \beta B I \right)$
Necrotic	$\frac{1}{c^*}\left( r E ( \frac{E + p B}{K}) + \gamma B ( \frac{q E + B}{K})\right)$

The drift term *f* in the SDE is then a linear combination of these forms. For example, when considering the total apoptotic biomarker levels, we can sum the two functional forms for baseline and evasive apoptotic tumors and use the resulting sum as the drift. The resulting drift equation is sum of a product of Lipschitz functions and as such, it is also Lipschitz.

Using this fact, we list some key properties of both diffusions. We first list the results for geometric case given by Eq. (3). Namely,

#### Theorem 3

(Existence and Uniqueness) Given that *E*, *B*, *I* are solutions of Eq. (1), the drift term *f* is a linear combination of the terms in Table 4 and consider the stochastic differential equation given by Eq. (3), then the solution exists, is unique almost surely, and given by

5 $$\begin{aligned} C(t) = C(0)&e^{\sigma \, W(t) - (\eta +\frac{1}{2} \sigma ^2) t} \nonumber \\&+ \int _0^t e^{\sigma \, \left( W(t) - W(s)\right) - (\eta +\frac{1}{2} \sigma ^2) (t - s)} f\left( E(s), B(s) , I(s) \right) \, ds. \end{aligned}$$

Furthermore, by the construction of *f* as sums and products of non-negative functions, *C*(*t*) is non-negative.

#### Proof

Section B.1.1 of the Appendix. $\square$

#### Theorem 4

(Mean and Variance) The mean and variance of the stochastic differential equation given by (3) is given by

6 $$\begin{aligned} m(t) = c_0 e^{-\eta t} + \int _0^t f(s) \,e^{-\eta (t - s) }ds \end{aligned}$$

and,

7 $$\begin{aligned} v_t =&c^2_0 e^{(\sigma ^2 - 2 \eta )t} + \int _0^t 2 e^{(\sigma ^2 - 2 \eta )(t - s)} m(s) f(s) ds - m(t)^2 \end{aligned}$$

respectively.

#### Proof

Section B.1.2 of the Appendix. $\square$

On the other hand, the square root diffusion given by Eq. (2) is a variant of the well-known Feller-type diffusion described in (Feller 1951; Karlin and Taylor 1975, 1981; Etheridge 2011) wherein, $\hat{\sigma } = \frac{\sigma }{\sqrt{c^*}}$ and we replace the constant migration term in Feller’s original formulation with a time-dependent production rate *f*(*t*) which yields an inhomogeneous square-root diffusion. In addition, we consider $\hat{f}(t) = f(t) + \xi$, where $\xi > \frac{\sigma ^2}{2}$ in order to satisfy the Feller condition for non-negativity. While we do not have an explicit path solution, we can obtain the mean and the variance. Due to the nature of the SDE, we find the mean of the process to be identical (with *f* replaced with $\hat{f}$) to the one described in Theorem 4.

#### Theorem 5

(Variance) The variance of the stochastic differential equation given by Eq. (2) is given by

8 $$\begin{aligned} v_t = c^2_0 e^{- 2 \eta t} + \int _0^t \left( 2 \hat{f}(s) +\hat{\sigma }^2 \right) e^{ - 2 \eta (t - s)} m(s) ds - m(t)^2. \end{aligned}$$

#### Proof

Section B.1.3 of the Appendix. $\square$

The above analytical characterization allows us to characterize the dynamics of biomarker release derived from the underlying tumor-immune interaction. Via Theorems 3, 4, and 5, we generate comprehensive trajectories for the representative equilibrium states and list them in Section S2 of the SI. Given a particular realization of the stochastic noise, we obtain a complete trajectory of the biomarker dynamics, capturing fluctuations, sharp transitions, and rare events that are often obscured in expectation-based analyses. These sample paths retain the full influence of volatility on the system — including its role in shaping the variability, extremes, and temporal structure of the output.

For the linear SDE case, we simulated both the path solutions obtained and the stochastic differential equation via different methods to validate the result. In particular, the SDE was numerically solved by the Euler-Maruyama scheme while the exact path solutions enable us to use Monte-Carlo simulations to generate the path trajectories. The root diffusion case was numerically solved by the Euler-Maruyama scheme as well. Furthermore, we use the mean and variance equations to generate the mean trajectories and the variance cone for both of the regimes of interest.

**Fig. 2:**
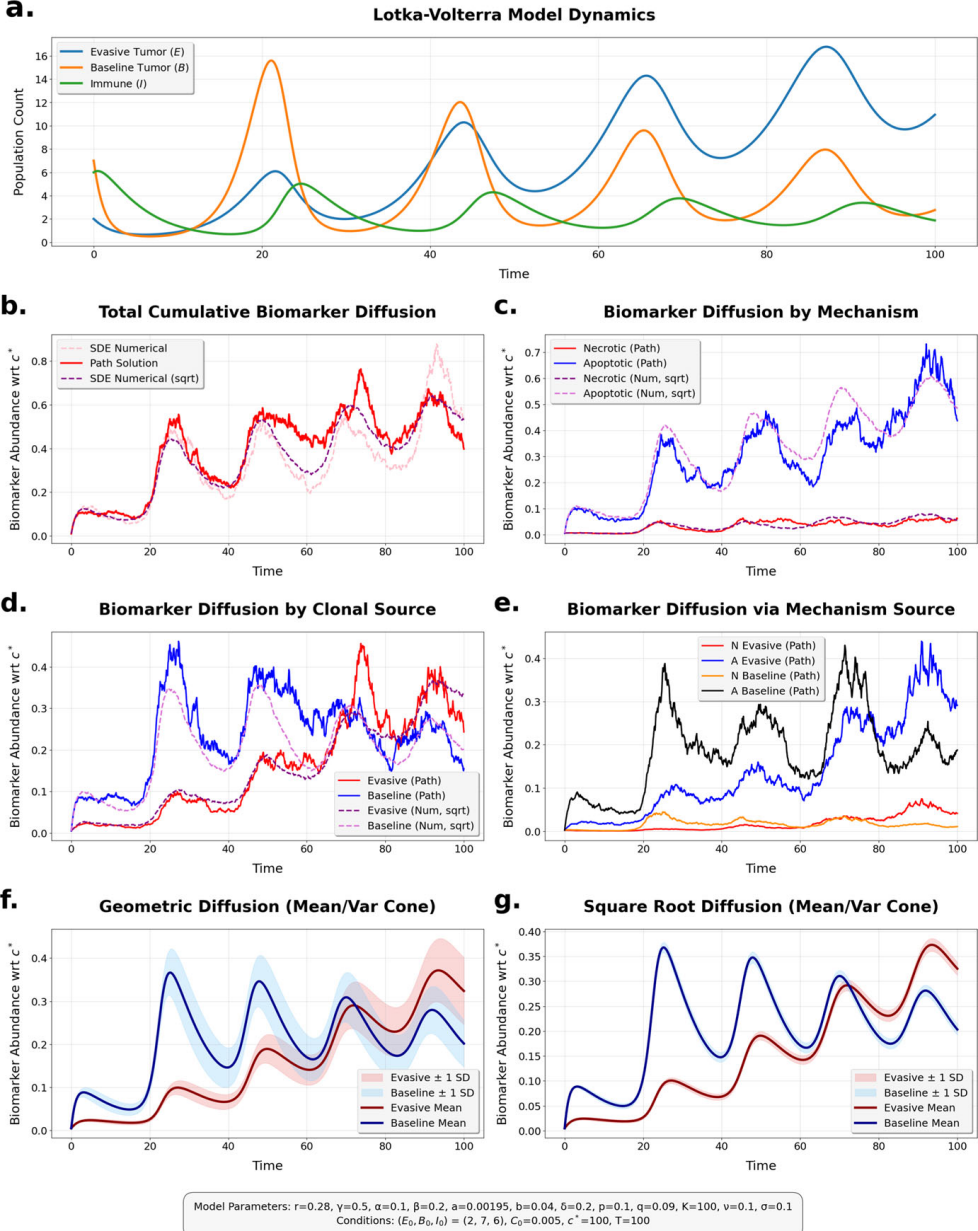
Dynamics of the heterogeneous immune mediated coexistence and associated biomarker release trajectories **a**) Lotka-Volterra Trajectory **b**) Cumulative biomarker trajectory **c**) Necrotic (N) vs. Apoptotic (A) Split **d**) Evasive vs Baseline Split **e**) Full table split **f**) Mean and Variance of the biomarker trajectories with geometric diffusion. **g**) Mean and Variance of the biomarker trajectories with square root diffusion. Dotted lines represent numerical simulations of (3) via Euler-Maruyama, lines represent path simulations of (5) via Monte Carlo simulations. In all cases, $r = .28, \gamma = 0.5, \alpha = 0.1, \beta = 0.2, a = 0.00195, b = 0.04, \delta = 0.2, p= 0.1 , q= 0.09, K = 100,\eta = 0.1 , \sigma = 0.1, (E_0,B_0,I_0) = (2,7,6) , C_0 = 0.005, c^* = 100$ were taken as model parameters

Tumor escape states with immune loss lead to a saturation of necrotic biomarker levels as the subpopulations overcrowd the shared carrying capacity (Figures S2c and S3c). On the other hand, immune-mediated coexistence generates a surplus of apoptosis-derived signal due to continued interactions between the adaptive immune and cancer compartments (Figures 2c, S1c). For our purposes, we provide a full analysis in this case, which involves all three populations (Figure 2). As the tumor approaches the interior equilibrium point, the population levels fluctuate in the predator-prey dynamics (Figure 2a). These dynamics subsequently generate a cumulative biomarker release trajectory (Figure 2b), which can be further partitioned by release mechanism (Figure 2c), cancer subpopulation (Figure 2d), or both (Figure 2e). In this particular example, we observe that heterogeneous immune mediated coexistence is driven primarily via T cell killing in this visualization, which represents cases where the tumor size is small with respect to the carrying capacity. Note that $c^*$ represents the minimal detection limit for the circulating biomarker. Under the model parametrization with $c^* = 100$ and equilibrium state $(E,B, I) = (2 , 7 , 6)$, the biomarker trajectory might not hit detection size at all. Consequently, we would expect such a tumor-immune interaction to provide minimal benefit to early detection, which might result in undetected cancer progression. Here, $c^*$ was chosen arbitrarily for foundational understanding.

#### Mean Time to Detection

The prior section demonstrated an example for which the cancer population is expected to fall below lower detection size for a significant amount of time. Since early time plays such a critical role in cancer diagnosis and treatment monitoring, we now look into time to detection. To accomplish this, we use the form of the stochastic differential equation given in Eqs. (2) and (3) alongside techniques from Itô theory to describe the expected time needed for the tumor to be detected under the dynamics for the LV model. We begin by stating the results for the linear SDE given by Eq. (3).

##### Lemma 6

(Backward equation for the mean detection time) Let

$$ \tau = \inf \{t>0:\, C(t)\ge 1\} $$

denote the first time the biomarker process reaches the detection threshold. For $(t,x)\in [0,\infty )\times (0,1)$, define the conditional mean time to detection by

$$ k(t,x) = \mathbb {E}[\tau - t \mid C_t = x]. $$

Then $k$ satisfies the backward Kolmogorov equation

9 $$\begin{aligned} \frac{\partial }{\partial t} k(t,x) + \big (f(t)-\eta x\big ) \, \frac{\partial }{\partial x}k(t,x) + \tfrac{1}{2} \sigma ^2 x^2 \, \frac{{\partial }^{2}}{\partial x^2}k_{xx}(t,x) = -1, \qquad (t,x)\in [0,\infty )\times (0,1), \end{aligned}$$

with boundary condition

10 $$\begin{aligned} k(t,1)=0, \qquad t\ge 0. \end{aligned}$$

Among all nonnegative functions $u$ satisfying (9)–(10), the mean detection time $k$ is the minimal one:

$$ k(t,x)\;\le \; u(t,x) \qquad \text {for all } (t,x)\in [0,\infty )\times (0,1). $$

In particular,

$$ \mathbb {E}[\tau \mid C(0)=c_0] = k(0,c_0). $$

##### Proof

Section B.1.3 of the Appendix. $\square$

Lemma 6 fully characterizes the mean detection time for general time-varying shedding profiles $f(t)$. To obtain a closed-form representation, we specialize to the biologically relevant regime in which the underlying tumor–immune dynamics have approached equilibrium and $f(t)\approx f^*$. In this case, the partial differential equation (PDE) reduces to an ODE. Namely,

##### Theorem 7

(Mean time to detection ($c^*$)) Let $\tau = \inf \{ t > 0 \, \vert \, C(t) \ge 1\}$ be a stopping time and let $f(t) \approx f^*$. Then the time to detection is given by

$$\begin{aligned} \mathbb {E}\left[ \tau \ \vert \ C(0) = c_0\right] = \sup _{x \in (0,1)}&\left( \frac{- \int _x^1 \int _x^v e^{-\int _v^y p(z) dz} dy \; q(v) dv}{\int _x^1 e^{\int _y^1 p(v) dv} dy } \right) \cdot \int _{c_0}^1 e^{\int _y^1 p(v) dv} dy\\&+ \int _{c_0}^1 \int _{c_0}^v e^{-\int _v^y p(z) dz} dy \; q(v) dv \end{aligned}$$

where,

11 $$\begin{aligned} p(x) = \frac{2(f^* - \eta x)}{\sigma ^2 x^2}, \quad q(x) =-\frac{2}{\sigma ^2 x^2}. \end{aligned}$$

##### Proof

Section B.1.3 of the Appendix. $\square$

We now pivot to the square root diffusion case given by Eq. (2). The mathematical framework and techniques for obtaining results like Lemma 6 and Theorem 7 are identical for both the SDE formulations, albeit with some minor differences. In particular, due to the nature of the square root diffusion as compared to the linear case, the backward Kolmogorov operator itself would have some minor changes that affect the result. Namely, the backward equation to solve will have the form

$$\begin{aligned} \frac{\partial }{\partial t} k(t,x) + \big (\hat{f}(t)-\eta x\big ) \frac{\partial }{\partial x}k(t,x) + \frac{1}{2}\hat{\sigma }^2 x \frac{{\partial }^{2}}{\partial x^2}k(t,x) = -1, \qquad (t,x)\in [0,\infty )\times (0,1). \end{aligned}$$

alongside the same boundary and probabilistic condition as in Lemma 6. Subsequently, we find that the form of the solution for the mean detection time is identical as in Theorem 7 with *p*(*x*) and *q*(*x*) changed to

12 $$\begin{aligned} \hat{p} = \frac{2 \big (\hat{f}^*-\eta x\big )}{\hat{\sigma }^2 x}, \quad \hat{q} =-\frac{2}{\hat{\sigma }^2 x}. \end{aligned}$$

**Fig. 3:**
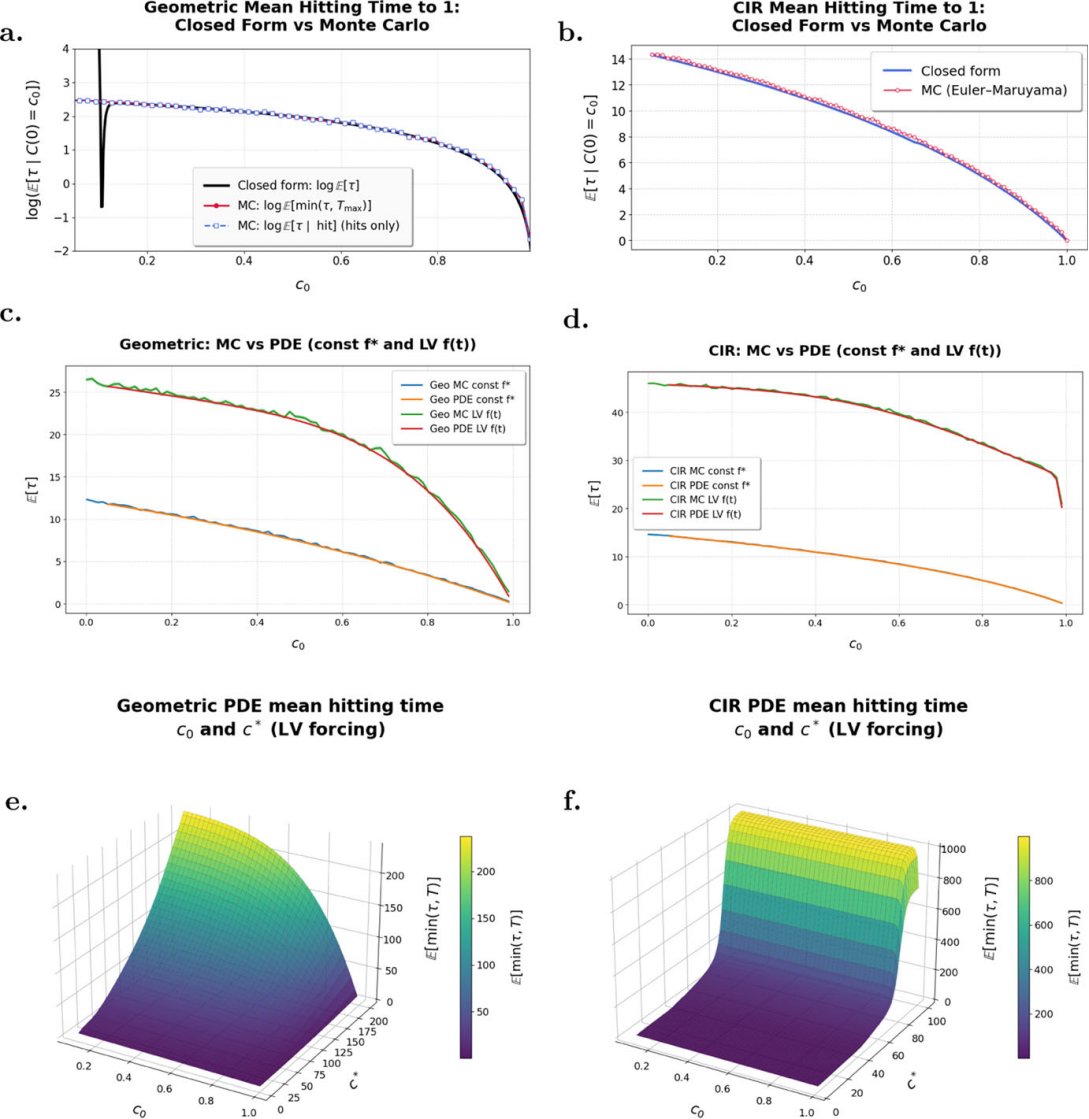
Comparison of hitting times for geometric and CIR diffusion. a,b) Closed form solution plotted against Monte-Carlo simulations. Despite numerical challenges at small $c_0$ values, we get good agreement with the theoretical and simulated hitting times. c,d) PDE and Monte-Carlo simulations for asymptotic vs longitudinal *f*(*t*) for the interior equilibrium case in both geometric and square-root (CIR) ctDNA diffusion models. We compare the PDE numerical solution with a Monte Carlo simulation. e,f) Parameter sweep for $c^*$: minimum detection limit for biomarker models. In all the plots, $r = 0.28, \gamma = 0.5, \alpha = 0.1, \beta = 0.2, a = 0.00195, b = 0.04, \delta = 0.2, p= 0.1 , q= 0.09, K = 100, \eta = 0.1 , \sigma = 0.25, (E_0,B_0,I_0) = (2,7,6) , C_0 = 0.1, C^* = 50$ were taken as model parameters

Although the expressions for mean hitting times are mathematically well-posed, their numerical evaluation proved unstable in practice. In particular, the supremum ratio appearing in both formulas frequently attained large values, leading to degenerate behavior for small initial conditions (Fig. 3a). Moreover, the closed-form expressions exhibited high sensitivity to parameter perturbations, limiting their robustness in computational studies. Importantly, these formulas are derived under equilibrium (time-independent) forcing *f*, and therefore do not directly accommodate longitudinal, time-varying dynamics. For these reasons, in both the CIR and geometric cases we computed the mean hitting time using the associated backward PDE formulation, imposing a finite regularity condition (Fig. 3c, d).

Under the interior-equilibrium parametrization of the Lotka–Volterra subsystem, the mean first-passage time to the biomarker detection threshold $c^*$ exhibited distinct sensitivity profiles across model parameters. Parameters that strengthened long-term tumor persistence, such as the evasive-clone growth rate *r* (Figs. S4c and S6c) or the immune decay rate $\delta$ (Figs. S4a and S6a), generally accelerated threshold crossing by sustaining larger tumor burdens and hence stronger biomarker forcing. In contrast, parameters that enhanced immune-mediated suppression or inter-subpopulation competition, including *a*, $\alpha$, $\beta$, and *p* (Figs S4-7), tended to delay detection by reducing the effective shedding source. Because the baseline parametrization satisfies $\alpha < \beta$, the baseline clone is more immune-sensitive than the evasive clone, so perturbations in $\alpha$ more directly suppress the long-term persistent tumor compartment and therefore exert a stronger influence on detection timing. Notably, several parameters, particularly *b* (Figs. S5b and S7b) and $\gamma$ (Figs. S4d and S6d), displayed non-monotone effects, indicating that detectability depends not only on net tumor burden but also on how ecological feedback reshapes the coexistence orbit and thus the time-dependent shedding force *f*(*t*).

To further evaluate the impact of clinical detection limits on threshold crossing, we explicitly varied the minimal detection signal $c^*$ alongside the initial biomarker concentration $c_0$. Because the normalized shedding force scales inversely with the detection limit $(f \propto 1/c^*)$, increasing $c^*$ simultaneously raises the detection boundary while suppressing the effective stochastic drift toward that boundary. In the geometric diffusion model (Fig. 3c), increasing $c^*$ produced a smooth and continuous extension of the expected time to detection, reflecting a gradual delay as assay sensitivity decreases. By contrast, the CIR formulation (Fig. 3d) exhibited a much sharper, threshold-like response. As the detection limit increased, a large region of parameter space expanded into an “evasion plateau”, where the expected hitting time saturated at the simulation horizon ($T=500$). Within this regime the Lotka–Volterra subsystem sustained an actively shedding tumor population $(f>0)$, yet the steady-state biomarker concentration remained confined below the assay’s sensitivity threshold. The abrupt “cliffs” surrounding this plateau mark the critical parameter boundary at which the deterministic shedding force finally overcomes the mean-reverting dynamics of the CIR process, triggering a rapid transition from effective long-term evasion to detectable biomarker levels. Across all parameter sweeps, the CIR biomarker model consistently exhibited sharper regime boundaries and larger saturation plateaus than the geometric model, suggesting that the square-root diffusion is more prone to transitions between parameter regimes with reliable threshold crossing and regimes where hitting becomes effectively unattainable within the observation horizon.

## Discussion

Challenges in tracking cancer dynamics near minimal detectable disease levels currently limit early diagnosis and identification of progression during disease modeling. To address this, we developed a general stochastic modeling framework to track the noisy biomarker release dynamics arising from a population undergoing cell turnover and immune-mediated apoptosis. Our model provides a theoretical framework for understanding biomarker trajectories that depend on different release mechanisms from the underlying tumor-immune interactions. We do so by coupling a system of ODEs describing heterogeneous tumor populations interacting with the immune system with SDEs describing biomarker trajectories.

In this study, we used a one-predator, two-prey competitive Lotka-Volterra model where the prey (tumor cells) compete with each other under a shared carrying capacity. We show the underlying ODE model allows for coexistence between immune-targeted and immune-evasive cancer subpopulations. Relative altruism and egoism between these subpopulations play a critical role in this model, which suggests that the dynamics of tumor escape are shaped not just by individual subpopulation fitness, but also by their relative influence on one another. This finding highlights the importance of cooperation and competition as fundamental forces in shaping disease heterogeneity following immune escape. For example, cooperative behavior, such as the shared secretion of growth factors, immune-modulating cytokines, or extracellular matrix remodeling enzymes, can enable certain subpopulations to persist despite being less fit in isolation (Marusyk et al. 2014). Conversely, competitive strategies, including resource monopolization or immune suppression, may allow one subpopulation to dominate but at the expense of overall tumor heterogeneity and adaptability (Parker et al. 2021; Keats et al. 2012). In our model, a single-population escape, for instance, is only stable if the competing subpopulation behaves altruistically, acting as a cooperative partner by sharing resources or by absorbing immune pressure without reciprocation. In contrast, immune escape and stable coexistence of immune-evasive and immune-targeted populations requires that both populations adopt the same strategy, either mutually egoistic or mutually altruistic. Otherwise, the egoistic subpopulation inevitably dominates, reducing heterogeneity to either an immune-evasive or targetable disease.

The complex interplay between model parameters and competitive dynamics is especially evident in the coexistence states. In the case of homogeneous immune-mediated coexistence, stability hinges on how these parameters align. Specifically, in (4), we derive a lower bound on the level of “assistance” required from the dying subpopulation for the dominant one to maintain balance with the immune system. This threshold depends on both the carrying capacity of the dominant subpopulation and the relative growth advantage between the two subpopulations. It effectively sets a minimum cooperation level needed to sustain equilibrium, highlighting how even a regressing subpopulation can play a stabilizing role in the tumor-immune landscape. This may imply that the extinction of a vulnerable subpopulation may reduce immune detection or competition for resources, indirectly supporting the survival of the more evasive subpopulation.

Recent empirical work has investigated such behavior in breast cancer (Masroni et al. 2023), wherein an altruistic subpopulation was observed to benefit the growth of the total population. These insights have potential implications for immunotherapy, as treatments that unintentionally eliminate the altruistic subpopulation may destabilize the system and pave the way for immune-resistant escape variants to expand unchecked. Additional experimental and modeling studies are needed to fully explore this therapeutic possibility.

Our model predicts that heterogeneous tumor escape requires additional constraints related to balancing the relative subpopulation fitness values with the predication dynamics of the immune system. Our results predict a critical bound in the competitive parameter that partitions cooperative and competitive cancer strategies, which suggests that subtle shifts in competition for cancer populations operating close to this bound can dramatically reshape tumor-immune dynamics. Biologically, this result suggests that cooperative behaviors among tumor cells, including shared production of growth factors or immune modulators, may be under selective pressure not just for their immediate benefit, but for their role in maintaining population heterogeneity during immune surveillance. Moreover, detecting such cooperation could offer early clues about impending immune escape in the setting of immune control, and disrupting these interactions could be one potential targeting strategy to destabilize the tumor ecosystem. Our findings echo broader ecological principles in evolutionary theory, where population stability depends on the payoff structure between cooperative and selfish strategies. In the context of cancer, this underscores that tumor progression is not merely a Darwinian race among isolated subpopulations, but also involves dynamic interactions between subpopulations exhibiting interdependent behavior (Masroni et al. 2025).

The principal strength of our framework is that it preserves the mathematical structure of the tumor–immune system while extending it to stochastic biomarker dynamics in a biologically interpretable way. Because the stochastic equations are directly coupled to tumor–immune interactions under shared carrying capacity, fluctuations in biomarker levels remain mechanistically grounded rather than purely phenomenological. This structure enables analytical study of stability, variance structure, and first-passage behavior while retaining biological transparency.

By incorporating both square-root (CIR-type) and multiplicative (geometric-type) stochastic formulations, we allow the variance structure of biomarker dynamics to vary across concentration regimes. The CIR formulation reflects count-limited variability and enforces non-negativity under suitable parameter conditions, whereas the geometric formulation captures proportional fluctuations and admits explicit pathwise solutions. These two representations should not be interpreted as competing biological hypotheses but as complementary approximations that emphasize different statistical features of biomarker noise. In particular, the geometric model produces lognormal-type variability and proportional variance growth at higher levels, while the CIR model yields gamma-like variability and may better reflect fluctuations when molecule counts are low. The choice of diffusion structure therefore directly influences tractability, variance scaling, and interpretation of inferred trajectories.

Our model represents heterogeneity through two subpopulations with different levels of immune targeting. While this abstraction captures the essential dynamics between more and less immune-targeted groups, real tumors span a broader distribution of immune recognition levels in addition to increasing clonal diversity. This simplification is compounded by the assumption that each SDE is driven by an independent Brownian motion, meaning fluctuations in one subpopulation’s biomarker shedding are unrelated to those in another. In practice, systemic influences could introduce correlated noise, potentially reshaping variance structure, modifying stability properties, and complicating parameter identifiability.

Tumor escape states, associated with immune evasion, are characterized by elevated necrotic biomarker levels, while immune-mediated coexistence yields a sustained but lower apoptotic signal due to ongoing tumor–immune interactions. However, only the escape dynamics consistently surpass critical detection thresholds, whereas the coexistence state often remains sub-threshold despite its biological activity (Figure 2e, f). This underscores a key limitation of detection strategies that rely solely on biomarker magnitude. Reinterpreting the boundary $c^*$ as an assay detection threshold provides insight into the stochastic nature of minimal residual disease monitoring. Our models demonstrate that when a tumor reaches an interior equilibrium with the immune system, it generates a constant, positive shedding force. However, if this steady-state signal rests below the assay’s sensitivity limit, the choice of noise structure dictates the statistics governing ultimate detection. The geometric model, with its proportional volatility, suggests that even sub-clinical steady states possess enough variance to occasionally spike across the detection threshold, yielding delayed but eventual discovery. In stark contrast, the CIR model’s square-root volatility and mean-reverting drift fiercely resist upward spikes when the system is trapped in the low-signal regime. This creates a “stealth plateau”—a rigid phase space where tumors persistently evade detection. From a translational perspective, these cliffs of tumor escape emphasize that linearly increasing an assay’s limit of detection (lowering $c^*$) does not merely linearly reduce time to diagnosis; in a CIR-governed biological environment, crossing that sensitivity threshold is physically required to break subclinical detection.

Overall, the framework provides a mathematically coherent bridge between mechanistic tumor–immune dynamics and empirically observed concentration-dependent variability in biomarker measurements. Extensions incorporating correlated stochastic drivers, richer clonal structure, or explicit observation frameworks may further enhance model application.

## Supplementary Information

Below is the link to the electronic supplementary material.

Supplementary file 1 (pdf 8646 KB)

## Data Availability

All data generated or analyzed during this study are included in this published article [and its supplementary information files].

## Appendix A Dynamical Behavior of the ODE

Let us consider the system of equations in Eq. (1). Namely,

A1 $$\begin{aligned} dE&= r E \left( 1 - \frac{E + p B}{K}\right) - \alpha E I, \nonumber \\ dB&= \gamma B \left( 1 - \frac{q E + B}{K}\right) - \beta B I , \nonumber \\ dI&= a E I + b B I - \delta I \end{aligned}$$

with $$p,q \in (0,1)$$ and $$E(0) , B(0), I(0) \ge 0$$.

### A.1 Existence/ Uniqueness

The right hand side of the system of equations given by Eq. (A1) are all continuous and Lipschitz on $\textbf{C}[0,T]$. Via the Picard-Lindeoff theorem we have the existence and uniqueness of the solutions in a small neighborhood around the initial conditions. Namely, there exists $\epsilon > 0$ such that the solution both exists and is unique on the interval $[t_0 - \epsilon , t_0 + \epsilon ]$. Since our system is Lipschitzian on [0, *T*], this result can be extended to the whole interval. Finally, the positive initial conditions imply that the solutions are non-negative.

### A.2 Proof for Theorem 1

Here we investigate the existence and stability of the system at the equilibrium states. We will focus in the interior equilibrium states for a major part of the analysis. The equilibrium states are the population values where the population levels stabilize:

A2 $$\begin{aligned} dE = dB = dI = 0. \end{aligned}$$

This implies that,

A3 $$\begin{aligned} r E \left( 1 - \frac{E + p B}{K}\right) - \alpha E I = 0 \iff E = 0 \text { or } I = \frac{r}{\alpha } \left( 1 - \frac{E + p B}{K}\right) . \end{aligned}$$

Similarly,

A4 $$\begin{aligned} \gamma B \left( 1 - \frac{q E + B}{K}\right) - \beta B I = 0 \iff B = 0 \text { or } I = \frac{\gamma }{ \beta } \left( 1 - \frac{qE + B}{K}\right) . \end{aligned}$$

Finally,

A5 $$\begin{aligned} a E I + b B I - \delta I = 0 \iff I = 0 \text { or } a E + b B = \delta . \end{aligned}$$

#### A.2.1 Boundary Equilibrium States

We can view the boundary states as extinction or dominance outcomes in this predator–prey interaction between the adaptive immune system and the tumor subpopulations. Clearly, the case where all populations are absent, (0, 0, 0), is the trivial equilibrium solution. If both the tumor compartments are zero, $E = B = 0$ then for the equilibrium conditions (A2) to hold, $I = 0$. As such, we next consider cases where one of the tumor subpopulations is not identically zero.

We first assume that the baseline tumor population at equilibrium is nonzero. Hence, $E = 0$ and $I = \frac{\gamma }{ \beta } ( 1 - \frac{qE + B}{K})$. We have two ways to satisfy (A2):

**Case 1:** $$I = 0$$

$$\begin{aligned} I = \frac{\gamma }{ \beta } \left( 1 - \frac{q E + B}{K}\right) =0 \iff B = K. \end{aligned}$$

Thus, the equilibrium state is at (0, *K*, 0) where the immune system and the evasive subpopulation are eliminated and the baseline recognizable subpopulation grows to carrying capacity.

**Case 2:** $$a E + b B = \delta $$

$$\begin{aligned} a E + b B = \delta \implies B = \frac{\delta }{b}. \end{aligned}$$

Using this value of *B*, we find that

$$ I = \frac{\gamma }{ \beta } \left( 1 - \frac{q E + B}{K}\right) = \frac{\gamma }{ \beta } \left( 1 - \frac{ \delta }{b K}\right) . $$

This gives us the equilibrium state

$$\left( 0 , \frac{\delta }{b}, \frac{\gamma }{ \beta } \left( 1 - \frac{ \delta }{b K}\right) \right) \text { with } K > \frac{\delta }{b}.$$

We can use the same logic to find the other two boundary equilibrium states for when the evasive cells out-compete the baseline cells ($B = 0$ and *E* nonzero). Finally, we look at the case where $I = 0$ with *E*, *B* are nonzero. Here,

$$\begin{aligned} 0 = \frac{r}{\alpha } \left( 1 - \frac{E + p B}{K}\right) \implies E + p B = K, \\ 0 = \frac{\gamma }{\beta } \left( 1 - \frac{qE + B}{K}\right) \implies qE + B = K. \end{aligned}$$

Solving the above system of equations, we obtain

$$\begin{aligned} E = \frac{K\left( 1-p\right) }{1-pq}, \qquad B =\frac{K\left( 1-q\right) }{1-pq} . \end{aligned}$$

Under these constraints for *p*, *q* , we find that $pq \ne 1$ and that the solutions for the tumor compartments are non-negative and bounded above by the carrying capacity. We note that for such an equilibrium condition to exist, the tumor subpopulations would need to be jointly egoistic ($0< p , q < 1$) or jointly altruistic ($p,q > 1$).

To summarize, we have the following boundary equilibrium states:

$$ \begin{aligned}&(0,0,0),\ (0,K,0),\ (K,0,0),\ \left( 0,\frac{\delta }{b},\frac{\gamma }{\beta }\!\left( 1-\frac{\delta }{bK}\right) \right) ,\\&\left( \frac{\delta }{a},0,\frac{r}{\alpha }\!\left( 1-\frac{\delta }{aK}\right) \right) ,\ \left( \frac{K(1-p)}{1-pq},\frac{K(1-q)}{1-pq},0\right) . \end{aligned} $$

#### A.2.2 Interior States

We pivot to the interior state where $$K> E ,B ,I > 0 $$ and (A2) holds. We have that

A6 $$\begin{aligned}&\frac{r}{\alpha } \left( 1 - \frac{E + p B}{K}\right) = \frac{\gamma }{ \beta } \left( 1 - \frac{qE + B}{K}\right) , \nonumber \\ \iff&\left( \gamma \alpha q - r\beta \right) E + \left( \gamma \alpha - r\beta p \right) B = K \left( \gamma \alpha - r\beta \right) ,\nonumber \\ \iff&\psi E + \phi B = \theta , \end{aligned}$$

where

$$\begin{aligned} \psi \triangleq \gamma \alpha q - r\beta , \quad \phi \triangleq \gamma \alpha - r\beta p, \quad \theta \triangleq K ( \gamma \alpha q - r\beta ). \end{aligned}$$

For such an interior solution to exist, $\phi$ and $\psi$ cannot be zero simultaneously. Thus,

A7 $$\begin{aligned} \gamma \alpha q \ne r\beta \text { and } \gamma \alpha \ne r\beta p \iff p \ne \frac{r \beta }{\gamma \alpha } \text { and } q \ne \frac{\gamma \alpha }{r \beta }. \end{aligned}$$

Interestingly, this implies a similar condition for existence as we obtained in the tumor coexistence case: $pq \ne 1.$ Indeed, if $p = q = 1$, the equilibrium conditions would imply that either at equilibrium, $I = 0$, which contradicts the interior solution case, or there exists a constant $k_0$ such that $\gamma \alpha = k_0 r \beta$. In the latter case, we find that $dE = k_0 dS$ which would mean that the evasive population is nothing but a scaled version of the baseline recognizable one. This also means that, under this very restrictive condition on the competitive rates, the two heterogeneous tumor populations can be modeled via cumulative tumor population given by $dT_{cum} = (1 + k_0) T_{cum}.$

We use Eq. (A6) alongside the fact that $aE + b B = \delta$ at the interior equilibrium state to obtain

A8 $$\begin{aligned} E = \frac{\delta \phi - \theta b}{\phi a - \psi b}, \quad B = \frac{a \theta - \delta \psi }{a \phi - b \psi }. \end{aligned}$$

The equilibrium state for *I* can be found via Eqs. (A3) or (A4). From our discussion on Section A.1, we have that the solution is positive and finite. As such, we can use the form of the equilibrium state to find conditions for existence of the solution.

- **Finite Solutions:** In addition to the criteria given by Eq. (A7), we have that
  $$\begin{aligned} \phi a - \psi b&\ne 0 \iff p \ne \frac{\gamma \alpha }{r \beta } + \frac{b}{a} \left( 1 - \frac{\gamma \alpha q}{r \beta }\right) . \end{aligned}$$
- **Well-Defined Solutions:** From the construction of the model itself, we have that $0< E,B < K$. First, we address non-negativity and then move to the boundedness condition. From Eq. (A8), $\delta \phi - \theta b$, $a \theta - \delta \psi$ , and $\phi a - \psi b$ all have the same sign. This provides an avenue by which we may analyze parametric regimes for the competition parameters *p* and *q*, thereby studying the effects of relative “egoism” and “altruism” in each cancer subpopulations. Without loss of generality, we assume all of the expressions to be negative with the understanding that the same calculations would apply for the other case but with an inequality switch. We look into the first term,
  A9 $$\begin{aligned} 0&> \delta \phi - \theta b = (\gamma \alpha - r \beta p ) \delta - K (\gamma \alpha - r \beta ) b ,\nonumber \\ \iff&p> \frac{\gamma \alpha }{r \beta } - \frac{bK }{\delta }\frac{\gamma \alpha }{r \beta } + \frac{bK }{ \delta }, \nonumber \\ \iff&p > 1 + \left( \frac{bK}{\delta } - 1 \right) \left( 1 - \frac{\gamma \alpha }{r \beta }\right) \triangleq p_0. \end{aligned}$$
  For the second term, we have
  A10 $$\begin{aligned} 0&> a \theta - \delta \psi = (\gamma \alpha - r \beta ) aK - \delta (\gamma \alpha q - r \beta ), \nonumber \\ \iff&q> \frac{r \beta }{\gamma \alpha } + \frac{aK }{\delta } - \frac{aK }{\delta } \frac{r \beta }{\gamma \alpha }, \nonumber \\ \iff&q > 1 +\left( \frac{aK}{\delta } - 1 \right) \left( 1 - \frac{r \beta }{\gamma \alpha }\right) \triangleq q_0. \end{aligned}$$
  Finally, for the last term, we get,
  A11 $$\begin{aligned} 0&> \phi a - \psi b = (\gamma \alpha - r \beta p ) a- (\gamma \alpha q - r \beta ) b, \nonumber \\ \iff&\frac{a}{b} \, p + \frac{\gamma \alpha }{r \beta }\, q \, >\, \frac{a}{b}\frac{\gamma \alpha }{r \beta } + 1 . \end{aligned}$$
  Summarizing the above discussion for the negative case, Eqs. (A9) and (A10) act as lower bounds for how helpful both cancer subpopulations need to be for the interior equilibrium state to exist. The model parameters allow one subpopulation to be more egoistic and the other more altruistic. Indeed, if the product of the death rate of the baseline recognized tumor and the growth rate of the evasive tumor is more than the product of the death rate of the evasive tumor and the growth rate of the baseline tumor, $r \beta > \gamma \alpha$, then $p_0> 1 > q_0$, then the relative net growth of the evasive tumor is greater than that of the baseline tumor. For this equilibrium point, the evasive tumor must necessarily be altruistic ( $p> p_0 > 1$), while the baseline recognized tumor is allowed a little bit of egoism ($q \in (q_0 , 1) \cup [1 , \infty )$). The reverse behavior can be obtained from the positive case as well. The third condition then puts a lower (or upper) bound for the joint contribution. We note here that the interior solution would not exist if one subpopulation is egoistic and one altruistic with respect to the $p_0$ and $q_0$ values. Now, we pivot to the immune compartment. From Eqs. (A3) and (A4), we have that at equilibrium,
  $$\begin{aligned} \frac{r}{\alpha K}(K - E - pB) = I = \frac{\gamma }{\beta K}(K - qE - B). \end{aligned}$$
  Thus, we can see that non-negativity for *I* requires that $$E + pB < K$$ and $$ qE + B <K$$. This condition alongside the fact that the carrying capacity condition imposes $$ E , B \le K$$, implies that there exists upper bound values for both *p*, *q*, say $$p_{max},q_{max}$$, independent of the $$p_0, q_0$$ values. In summary, for the interior state to to exist, we would need $$p< p_{max}, q < q_{max}$$ and one of the following to hold,
  $$ (p>p_0 \ \text {and}\ q>q_0) \quad \text {or} \quad (p<p_0 \ \text {and}\ q<p_0). $$

#### A.2.3 Local Stability

The system of ODE’s described by Eq. (A1) is nonlinear. One way to check stability is to linearize the model around the equilibrium states. To perform this linearization, we calculate the Jacobian matrix by taking the partial derivatives of the system’s equations with respect to each variable. These partial derivatives form part of the Jacobian matrix, which represents the linearized system near an equilibrium state. Analyzing the eigenvalues of this Jacobian allows us to assess the local stability of the equilibrium. From the Hartman-Grobman Theorem, we have that near a hyperbolic equilibrium state (one where the Jacobian has no eigenvalues with zero real parts), the behavior of the nonlinear system is topologically equivalent to its linearized version (Perko 2012). Taking the partials of the system given by (A1), we get

A12 $$\begin{aligned} f (E,B,I)&\triangleq r E \left( 1 - \frac{E + p B}{K}\right) - \alpha E I ,\nonumber \\ g (E,B,I)&\triangleq \gamma B \left( 1 - \frac{q E + B}{K}\right) - \beta B I ,\nonumber \\ h (E,B,I)&\triangleq a E I + b B I - \delta I. \end{aligned}$$

Then the Jacobian of the system is given by

$$ J = \begin{bmatrix} r \left( 1 - \frac{2 E + p B }{K}\right) -\alpha I & - \frac{r p E}{K} & -\alpha E \\ - \frac{\gamma q B}{K} & \gamma \left( 1 - \frac{q E + 2 B }{K}\right) -\beta I & -\beta B \\ a I & bI & aE + b B - \delta \end{bmatrix}. $$

**Boundary Equilibrium States:**

- $$e_0 = (0,0,0):$$
  $$J \Big \vert _{e_0} = \begin{bmatrix} r & 0 & 0 \\ 0 & \gamma & 0 \\ 0 & 0 & - \delta \end{bmatrix} . $$
  The Jacobian at $e_0$ has eigenvalues $r > 0$, $\gamma > 0$, and $-\delta < 0$, indicating that this state is unstable. The positive eigenvalues for the evasive and baseline tumor subpopulations imply that near zero population, both tumor subpopulations will grow rather than die off. The negative eigenvalue for the immune compartment indicates that the immune system is suppressed in this region and is an artifact of the model rather than a biological reality. This limit is largely outside of the outcomes of interest, wherein at least one of the tumor subpopulations and immune systems dominate.
- **(Baseline tumor prevails)** $e_1 = (0, K , 0):$
  $$J \Big \vert _{e_1} = \begin{bmatrix} r \left( 1 - p\right) & 0 & 0 \\ - \gamma q & -\gamma & -\beta K\\ 0 & 0 & b K - \delta \end{bmatrix} $$
  The Jacobian at $e_1$ has eigenvalues $\lambda = -\gamma , r \left( 1 - p\right) , b K - \delta$. Here, $-\gamma < 0$, meaning that the local stability of the case where the baseline tumor prevails depends the signs of the other two eigenvalues. Thus, $e_1$ is stable when the equilibrium value of the baseline tumor population is more than the carrying capacity and the evasive tumor population is altruistic. **Stability Condition:** $K < \frac{\delta }{b}$ and $p > 1$.
- **(Evasive tumor prevails)** $$e_2 = (K, 0, 0): $$
  $$J \Big \vert _{e_2} = \begin{bmatrix} -r & -rp & -\alpha K \\ 0& \gamma \left( 1 - q\right) & 0 \\ 0 & 0 & aK - \delta \end{bmatrix}. $$
  The Jacobian at $e_1$ has eigenvalues $\lambda = -r ,\,\gamma \left( 1 - q\right) ,\, aK - \delta$. Thus, $e_2$ is stable when the equilibrium value of the evasive tumor population is less than the carrying capacity and the baseline tumor population is altruistic. **Stability Condition:** $K < \frac{\delta }{a}$ and $q > 1$.
- **(Evasive tumor eliminated, baseline tumor coexists with the immune compartment)** $e_3 = \left( 0 , \frac{\delta }{b}, \frac{\gamma }{ \beta } \left( 1 - \frac{ \delta }{b K}\right) \right) :$
  $$ J \Big \vert _{e_3} = \begin{bmatrix} r \left( 1 - \frac{ p \delta }{bK}\right) -\frac{\gamma \alpha }{ \beta } ( 1 - \frac{ \delta }{b K}) & 0 & 0 \\ - \frac{\gamma q \delta }{bK} & - \frac{ \gamma \delta }{bK} & -\beta \frac{\delta }{b} \\ \frac{a\gamma }{ \beta } ( 1 - \frac{ \delta }{b K}) & \frac{b \gamma }{ \beta } ( 1 - \frac{ \delta }{b K}) & 0 \end{bmatrix}. $$
  The Jacobian at $e_3$ has eigenvalues
  $$ \lambda = r \left( 1 - \frac{ p \delta }{bK}\right) -\frac{\gamma \alpha }{ \beta } \left( 1 - \frac{ \delta }{b K}\right) $$
  and $\lambda _{\pm }$ which satisfies the quadratic equation,
  $$\lambda ^2 + \frac{ \gamma \delta }{bK} \lambda + \delta \gamma \left( 1 - \frac{ \delta }{b K}\right) = 0.$$
  For the quadratic equation to have negative real parts, we use the Routh-Hurwitz Stability criterion (Anagnost and Desoer 1991) for quadratic polynomials which states that a monic quadratic equation will have negative real roots if and only if the coefficients are positive. This holds true as the baseline tumor equilibrium value is less than the carrying capacity for this equilibrium state. Finally, we need
  $$\begin{aligned} r \left( 1 - \frac{ p \delta }{bK}\right)&< \frac{\gamma \alpha }{ \beta } \left( 1 - \frac{ \delta }{b K}\right) , \\ \iff \delta (\gamma \alpha - r \beta p)&< bK(\gamma \alpha - r \beta ) . \end{aligned}$$
  This is nothing but $\delta \phi - \theta b < 0$. Thus, from our interior equilibrium calculation in Eq. (4), we find that the equilibrium state where the evasive tumor dies out while the baseline and the immune compartment coexist imposes a lower bound to how altruistic the the evasive population can be. Namely, we get the following stability criterion. **Stability Criterion: **
  $$ \frac{\delta }{b} < K, \qquad p > 1 + \left( \frac{bK}{\delta }-1\right) \!\left( 1-\frac{\gamma \alpha }{r\beta }\right) . $$
- **(Baseline tumor eliminated, evasive tumor coexists with the immune compartment)** $e_4 = (\frac{\delta }{a} , 0, \frac{r}{ \alpha } ( 1 - \frac{ \delta }{a K})):$
  $$J \Big \vert _{e_4} = \begin{bmatrix} - \frac{r\delta }{aK} & - \frac{r p \delta }{aK} & -\alpha \frac{\delta }{a} \\ 0 & \gamma \left( 1 - \frac{q \delta }{aK}\right) -\frac{r \beta }{ \alpha } ( 1 - \frac{ \delta }{a K}) & 0 \\ a \frac{r}{ \alpha } ( 1 - \frac{ \delta }{a K}) & \frac{rb}{ \alpha } ( 1 - \frac{ \delta }{a K}) & 0 \end{bmatrix} . $$
  With a similar argument to that of the previous case, we can say that the stability at this equilibrium value depends on the sign of the eigenvalue given by
  $$\lambda = \gamma \left( 1 - \frac{q \delta }{aK}\right) -\frac{r \beta }{ \alpha } \left( 1 - \frac{ \delta }{a K}\right) , $$
  and the roots of the following quadratic equation
  $$ \lambda ^2 + \frac{ r \delta }{aK} \lambda + \delta r ( 1 - \frac{ \delta }{a K}) = 0. $$
  Similar to the symmetric case where the baseline coexists with immune compartment, we have that
  $$\begin{aligned} \gamma \left( 1 - \frac{q \delta }{aK}\right) -\frac{r \beta }{ \alpha } \left( 1 - \frac{ \delta }{a K}\right)< 0 \iff a \theta - \delta \psi < 0. \end{aligned}$$
  Using the result of Eq. (A10), we obtain a lower bound for how altruistic the baseline tumor needs to be in order for the evasive tumor to eliminate it and coexist with the immune compartment. **Stability Criterion: **
  $$ \frac{\delta }{a} < K, \qquad q > 1 + \left( \frac{aK}{\delta }-1\right) \!\left( 1-\frac{r\beta }{\gamma \alpha }\right) . $$
- **(Immune compartment eliminated, tumor co-existence)** $e_5 \!=\! \left( \frac{K\left( 1-p\right) }{1-pq} ,\right. \left. \frac{K\left( 1-q\right) }{1-pq} , 0\right)$
  $$J \Big \vert _{e_5} = \begin{bmatrix} \frac{ -r E }{K} & - \frac{r p E}{K} & -\alpha E \\ - \frac{\gamma q B}{K} & \frac{ - \gamma B }{K} & -\beta B \\ 0 & 0 & aE + b B - \delta \end{bmatrix}. $$
  The Jacobian at $e_5$ has eigenvalues given by
  $$\lambda = \delta - aE - b B $$
  and the roots of the following quadratic equation
  $$ \lambda ^2 + \frac{ r (1- p) + \gamma (1-q) }{1 - pq } \lambda + r \gamma \frac{(1-p)(1-q)}{1 - pq } = 0. $$
  From earlier discussion in Sec. A.2.1, we have that the tumor co-existence equilibrium requires both tumor subpopulations to simultaneously be either both egoistic or both altruistic. **Stability Criterion: **
  $$ (p,q<1 \ \text {or}\ p,q>1), \qquad \delta > \frac{K}{1-pq}\big (a(1-p)+b(1-q)\big ). $$
- **Interior Equilibrium State:** The Jacobian for interior equilibrium state is given by:
  $$ J \Big \vert _{e_6} = \begin{bmatrix} -\frac{r}{ K} \frac{\delta \phi - \theta b}{\phi a - \psi b} - \lambda & - \frac{r p}{K}\frac{\delta \phi - \theta b}{\phi a - \psi b} & -\alpha \frac{\delta \phi - \theta b}{\phi a - \psi b} \\ - \frac{\gamma q }{K} \frac{a \theta - \delta \psi }{a \phi - b \psi } & -\frac{\gamma }{ K} \left( \frac{a \theta - \delta \psi }{a \phi - b \psi } \right) - \lambda & -\beta \frac{a \theta - \delta \psi }{a \phi - b \psi } \\ a \frac{r}{\alpha K} \left( K -\frac{\delta \phi - \theta b}{\phi a - \psi b} - p \frac{a \theta - \delta \psi }{a \phi - b \psi } \right) & b \frac{\gamma }{\beta K} \left( K - q\frac{\delta \phi - \theta b}{\phi a - \psi b} - \frac{a \theta - \delta \psi }{a \phi - b \psi } \right) & a \frac{\delta \phi - \theta b}{\phi a - \psi b} + b \frac{a \theta - \delta \psi }{a \phi - b \psi } - \delta - \lambda \end{bmatrix} $$
  with the characteristic functions given by
  $$\begin{aligned} \lambda ^3 + A \lambda ^2 + B \lambda + C \end{aligned}$$
  where,
  $$\begin{aligned} A =&\frac{r \hat{E}}{K} + \frac{\gamma \hat{B}}{K} - a \hat{E} - b \hat{B} +\delta , \\ B =&\frac{r \gamma \hat{E} \hat{B}}{K^2} - \frac{r a \hat{E}^2}{K} - \frac{r b \hat{E} \hat{B}}{K} + \frac{r \delta \hat{E}}{K} - \frac{\gamma a \hat{E} \hat{B}}{K} - \frac{\gamma b \hat{B}^2}{K} + \frac{\gamma \hat{B} \delta }{K} + b \gamma \hat{B} - \frac{b \gamma q \hat{E} \hat{B}}{K} - \frac{b \gamma \hat{B}^2}{K} \\&-\frac{r p \gamma q \hat{E} \hat{B} }{K^2} + a r \hat{E} - \frac{a r \hat{T}^2_1}{K} - \frac{arp\hat{E} \hat{B} }{K^2} , \\ C =&\left( - \frac{r \gamma a \hat{E}^2 \hat{B}}{K^2}- \frac{r \gamma b \hat{E} \hat{B}^2}{K^2} + \frac{r \gamma \delta \hat{E} \hat{B}}{K^2} + \frac{r b \gamma \hat{E} \hat{B}}{K} - \frac{r b \gamma q \hat{E}^2 \hat{B}}{K^2} - \frac{r b \gamma \hat{E} \hat{B}^2}{K^2} \right) \\&+ \left( \frac{r p \gamma q a \hat{E}^2 \hat{B}}{K^2} + \frac{r p \gamma q b \hat{E} \hat{B}^2}{K^2} - \frac{r p \gamma q \delta \hat{E} \hat{B}}{K^2} - \frac{a \beta r^2 p \hat{E} \hat{B}}{\alpha K} + \frac{a \beta r^2 p \hat{E}^2 \hat{B}}{\alpha K^2} + \frac{a \beta r^2 p^2 \hat{E} \hat{B}^2}{\alpha K^2} \right) \\&- \alpha \hat{E} \left( \frac{b \gamma ^2 q \hat{B}}{\beta K} - \frac{b \gamma ^2 q^2 \hat{E} \hat{B}}{\beta K^2} - \frac{b \gamma ^2 q \hat{B}^2}{\beta K^2} - \frac{a\gamma r \hat{B}}{\alpha K} + \frac{a\gamma r \hat{E} \hat{B}}{\alpha K^2} + \frac{a\gamma r p \hat{B}^2}{\alpha K^2} \right) , \end{aligned}$$
  where
  $$\begin{aligned} \hat{E} = \frac{\delta \phi - \theta b}{\phi a - \psi b}, \qquad&\hat{B} = \frac{a \theta - \delta \psi }{a \phi - b \psi }. \end{aligned}$$
  Hence, from the Routh-Hurwitz stability criterion (Anagnost and Desoer 1991), we have that the above expression will have negative real part in their eigenvalues if and only if the following inequalities hold for the above coefficients:
  $$ A,C>0, \qquad AB - C > 0. $$

The above discussion about existence and local stability of equilibrium states is summarized in Theorem 1 in the main text.

### A.3 Proof for Theorem 2

**Global Stability:**

In section A.2.3, we described stability in a local sense. Namely, that the stability analysis via the linearized system only describes the behavior of trajectories close to the equilibrium, not their behavior far from it. In contrast, global stability concerns the stability of the system across the entire state space, not just near specific equlibria. Now we look into global stability for the interior equilibrium state. We employ Lyapunov’s second method of stability to find conditions for global stability. Consider the following functional given by

A13 $$\begin{aligned} L(E, B ,I) = P \left( E - \hat{E} - \hat{E} \ln {\frac{E}{ \hat{E}}} \right) + Q \left( B - \hat{B} - \hat{B} \ln {\frac{B}{ \hat{B} }} \right) + R \left( I - \hat{I} - \hat{I} \ln {\frac{I}{ \hat{I} }} \right) \end{aligned}$$

where *P*, *Q*, *R* are positive constants to be chosen later. Here, $L(\hat{E} , \hat{B} , \hat{I} ) = 0$ and using the fact that, $\ln {x} \ge x - 1$ for $x > 0$,

$$\begin{aligned} P \left( E - \hat{E} - \hat{E} \ln {\frac{E}{ \hat{E}}} \right)&\ge P \left( E - \hat{E} + \hat{E} \left( 1- \frac{E}{ \hat{E}} \right) \right) =0. \end{aligned}$$

Hence, $L(E, B ,I) \ge 0.$. Now we differentiate *L* with respect to time to get

$$\begin{aligned} \frac{dL}{dt}&= P \left( \frac{E - \hat{E}}{E} \right) \frac{dE}{dt} + Q \left( \frac{B - \hat{B}}{B}\right) \frac{dB}{dt} + R \left( \frac{I - \hat{I}}{I}\right) \frac{dI}{dt} \triangleq I + II + III . \end{aligned}$$

For convenience, lets focus on each part separately.

$$\begin{aligned} I&= P \left( \frac{E - \hat{E}}{E} \right) \frac{dE}{dt} \\&= P \left( E - \hat{E}\right) \left( r \left( 1 - \frac{E + p B}{K}\right) - \alpha I\right) \\&= P \left( E - \hat{E}\right) \left( r \left( 1 - \frac{E + p B}{K}\right) - \alpha I - r \left( 1 - \frac{\hat{E} + p \hat{B}}{K}\right) + \alpha \hat{I} \right) \\&= - \frac{rP}{K} \left( E - \hat{E}\right) ^2 - \frac{prP}{K} \left( E - \hat{E} \right) \left( B - \hat{B}\right) - \alpha P \left( I - \hat{I} \right) \left( E - \hat{E} \right) . \end{aligned}$$

$$\begin{aligned} II= &  Q \frac{B - \hat{B}}{B} \frac{dB}{dt}\\= &  Q \left( B - \hat{B}\right) \left( \gamma \left( 1 - \frac{q E + B}{K}\right) - \beta I \right) \\= &  Q \left( B - \hat{B}\right) \left( -\frac{\gamma q}{K} \left( E - \hat{E}\right) - \frac{\gamma }{K} \left( B - \hat{B}\right) - \beta \left( I - \hat{I} \right) \right) \\= &  -\frac{\gamma qQ}{K} \left( B - \hat{B}\right) \left( E - \hat{E}\right) - \frac{\gamma Q}{K} \left( B - \hat{B}\right) ^2 - \beta Q \left( I - \hat{I} \right) \left( B - \hat{B}\right) . \end{aligned}$$

$$\begin{aligned} III&= R \frac{I - \hat{I}}{I} \frac{dI}{dt} \\&= R\left( I - \hat{I} \right) \left( a E + b B - \delta \right) \\&= aR\left( I - \hat{I} \right) \left( E - \hat{E}\right) + b R\left( I - \hat{I} \right) \left( B - \hat{B}\right) . \end{aligned}$$

Thus,

$$\begin{aligned}\displaystyle I + II + III&= - \frac{rP}{K} \left( E - \hat{E}\right) ^2 - \frac{\gamma Q}{K} \left( B - \hat{B}\right) ^2 \\&\quad - \left( \frac{Prp}{K} +\frac{\gamma qQ}{K} \right) \left( E - \hat{E} \right) \left( B - \hat{B}\right) \\&\quad + \left( aR - \alpha P \right) \left( I - \hat{I} \right) \left( E - \hat{E}\right) \\&\quad + \left( b R - \beta Q \right) \left( I - \hat{I} \right) \left( B - \hat{B}\right) . \end{aligned}$$

If we select $P = p$, $R = \frac{\alpha p}{a}$, and $Q = \frac{b \alpha p}{ a \beta }$ then the above expression can be simplified to

$$\begin{aligned}\scriptstyle I + II + III&= - \frac{rp}{K} \left( E - \hat{E}\right) ^2 - \frac{\gamma b \alpha p}{a \beta K} \left( B - \hat{B}\right) ^2 \\&\quad - \left( \frac{rp^2}{K} +\frac{\gamma q b \alpha p}{ a \beta K} \right) \left( E - \hat{E} \right) \left( B - \hat{B}\right) . \end{aligned}$$

This means that, $\frac{dL}{dt} < 0$ when either of the following conditions hold:

$$ (E>\hat{E} \ \text {and}\ B>\hat{B}) \quad \text {or} \quad (E<\hat{E} \ \text {and}\ B<\hat{B}). $$

Thus, if the above conditions hold, *L* is a Lyapunov function and the interior state is asymptotically stable in Lyapunov’s sense. This means that trajectories that have the evasive and baseline tumor population jointly above or below the equilibrium state will stably converge to the interior equilibrium state asymptotically. The above discussion about global stability of interior equilibrium state is summarized in Theorem 2 in the main text.

## Appendix B Dynamical Behavior of the SDE

Let *C*(*t*) denote the biomarker concentration in blood, normalized by the minimal burden $c^*$ required for liquid biopsy detectability, and let $t \in [0,T]$. In the moderate- and high-count regimes—where both biological and measurement variability scale proportionally with concentration (Henriksen et al. 2022; Ye et al. 2025)—we represent ctDNA dynamics with the SDE

B14 $$\begin{aligned} dC = \left[ f(E,B,I) - \eta C \right] dt + \sigma C \, dW(t), \end{aligned}$$

where *W*(*t*) is a standard Brownian motion. In contrast, when biomarker concentrations fall near the limit of detection, we instead use a $\sqrt{C}$ diffusion term,

B15 $$\begin{aligned} dC = \left[ f(E,B,I) - \eta C \right] dt + \hat{\sigma } \sqrt{C} \, dW(t), \end{aligned}$$

where $$ \hat{\sigma } = \frac{\sigma }{\sqrt{c^*}}$$.

For foundational understanding, let us motivate the functional forms of the drift term by interpreting ctDNA to be the biomarker to model. Under that assumption, we consider the possible forms and rationale for the drift term *f* given by Table 4.

### B.1 Properties of the Solution of the Geometric SDE

#### B.1.1 Proof for Theorem 3

We have that that *E* and *B* are both bounded via the logistic growth term. This implies that $\exists \, I \in [0, \infty )$ such that all the derivatives given by Eq.(A1) are all negative. As such, *I* is also upper bounded in the interval [0, *T*]. Since all the derivatives are bounded, we have that the functions, *E*, *B*, and *I* are Lipschitz in time. As sums of products of Lipschitz functions, *f* is Lipschitz in time. Thus, the unique solution of the system of SDEs given by B14 with $E(0),B(0) , I(0) > 0$ and $C(0) > 0$ both exists (Theorem 11.1.1 of Kuo (2006)) and given by

$$\begin{aligned} C(t) = C(0)&e^{\sigma \, W(t) - (\eta +\frac{1}{2} \sigma ^2) t} \\&+ \int _0^t e^{\sigma \, \left( W(t) - W(s)\right) - (\eta +\frac{1}{2} \sigma ^2) (t - s)} f\left( E(s), B(s) , I(s) \right) \, ds. \end{aligned}$$

Furthermore, by the construction of *f* as sums and products of non-negative functions, *f* itself is non-negative. This implies that $C(t) \ge 0$. The above discussion regarding existence and non-negativity of the geometric SDE is summarized in Theorem 3 in the main text.

#### B.1.2 Proof for Theorem 4

Let us consider the class of stochastic differential equations given by

B16 $$\begin{aligned} dC(t)&= \left[ f(t) - \eta C(t) \right] dt + \sigma C(t) \, dW(t), \quad C(0) = c_0 > 0. \end{aligned}$$

**Mean: **Let $m(t) = \mathbb {E}\left[ C(t)\right]$. Then *m*(*t*) satisfies the ordinary differential equation given by

$$ \frac{dm(t)}{dt} = f(t) - \eta m(t), \quad m(0) = c_0. $$

This is a first-order linear ordinary differential equation. Thus, the mean of Eq (B16) can be explicitly written as

B17 $$\begin{aligned} m(t) = c_0 e^{-\eta t} + \int _0^t f(s) \,e^{-\eta (t - s) }ds. \end{aligned}$$

**Variance: ** Consider $h(x) = x^2$. Using the Itô formulae on *h*(*C*), we have

$$\begin{aligned} dh(C) = h^\prime (C) dC + \frac{1}{2} h^{\prime \prime }(C) (dC)^2 = \left[ 2 C f(t) - 2 \eta C^2 + \sigma ^2 C^2 \right] dt + 2 \sigma C^2 dW(t). \end{aligned}$$

Taking the expectation of both sides and using the fact that the Itô integral is zero mean, we have that

$$\begin{aligned} \frac{d\mathbb {E}\left[ C(t)^2\right] )}{dt} = 2 \mathbb {E}\left[ C(t)\right] f(t) - 2 \eta \mathbb {E}\left[ C(t)^2\right] + \sigma ^2 \mathbb {E}\left[ C(t)^2\right] . \end{aligned}$$

Let $v(t) =\mathbb {E}\left[ C(t)^2\right]$ then the second moment of the stochastic differential equation given by Eq (B16) solves the ODE given by

$$\begin{aligned} \frac{dv(t)}{dt} = 2 m(t) f(t) +(\sigma ^2- 2 \eta ) v(t), \quad v_0 = c_0^2. \end{aligned}$$

Noting that the above equation is a linear first order equation, we can use the integrating factor, $e^{-(\sigma ^2 - 2 \eta )t}$. Multiplying both sides of the above equation, we get

$$\begin{aligned} e^{-(\sigma ^2 - 2 \eta )t} \frac{dv(t)}{dt} -(\sigma ^2- 2 \eta ) e^{-(\sigma ^2 - 2 \eta )t} v(t)&= 2 m(t) f(t) e^{-(\sigma ^2 - 2 \eta )t} ,\\ \frac{d}{dt} \left( e^{-(\sigma ^2 - 2 \eta )t} v(t) \right)&= 2 m(t) f(t) e^{-(\sigma ^2 - 2 \eta )t}. \end{aligned}$$

Thus,

$$\begin{aligned} v(t)&= c^2_0 e^{(\sigma ^2 - 2 \eta )t} + \int _0^t 2 e^{(\sigma ^2 - 2 \eta )(t - s)} m(s) f(s) ds \\&= c^2_0 e^{(\sigma ^2 - 2 \eta )t} + \int _0^t 2 e^{(\sigma ^2 - 2 \eta )(t - s)} \left( c_0 e^{-\eta s} + \int _0^s f(u) \,e^{-\eta (s - u) } du\right) f(s) ds . \end{aligned}$$

Thus, variance can be found to be

$$\begin{aligned} Var(C(t))&= v(t) - m(t)^2\\&= c^2_0 e^{(\sigma ^2 - 2 \eta )t} + \int _0^t 2 e^{(\sigma ^2 - 2 \eta )(t - s)} m(s) f(s) ds - m(t)^2 \\&= c^2_0 e^{(\sigma ^2 - 2 \eta )t} + \int _0^t 2 e^{(\sigma ^2 - 2 \eta )(t - s)} \left( c_0 e^{-\eta s} + \int _0^s f(u) \,e^{-\eta (s - u) } du\right) f(s) ds \\&\quad - \left( c_0 e^{-\eta t}+ \int _0^t f(s) \,e^{-\eta (t - s) } ds \right) ^2. \end{aligned}$$

The above discussion about mean and variance of the geometric SDE is summarized in Theorem 4 in the main text.

#### B.1.3 Proof for Lemma 6 and Theorem 7

Let $\tau = \inf \{ t > 0 \, \vert \, C(t) \ge 1\}$ be a stopping time. This represents the expected time for the biomarker levels to reach detection levels given underlying tumor dynamics. Let us first consider the mean hitting time problem for the general case with a time dependent *f*(*t*). We start with the SDE given by Eq. (B16). For $(t,x)\in [0,\infty )\times (0,1)$, define the conditional mean time to detection by

$$\begin{aligned} k(t,x) = \mathbb {E}\left[ \tau - t \ \vert \ C_t = x\right] . \end{aligned}$$

In particular, when $C_0 = c_0 \in (0,1)$ we have

$$\begin{aligned} \mathbb {E}\left[ \tau \ \vert \ C_0 = c_0\right] = k(0,c_0). \end{aligned}$$

To derive the backward equation for *k*, we apply Itô’s lemma to the process $k(t,C_t)$:

$$\begin{aligned} dk(t,C_t)&= \partial _t k(t,C_t)\,dt + k_x(t,C_t)\,dC_t + \frac{1}{2}k_{xx}(t,C_t)(dC_t)^2 \\&= \Big [ \partial _t k(t,C_t) + \big (f(t)-\eta C_t\big ) k_x(t,C_t) + \tfrac{1}{2} \sigma ^2 C_t^2 k_{xx}(t,C_t) \Big ]dt + \sigma C_t k_x(t,C_t)dB_t . \end{aligned}$$

Integrating from 0 to $\tau$ and using that $k(\tau ,1)=0$ by definition of $\tau$, we obtain

$$\begin{aligned} -k(0,c_0)&= \int _0^\tau \Big [ \partial _t k(t,C_t) + \big (f(t)-\eta C_t\big ) k_x(t,C_t) + \tfrac{1}{2} \sigma ^2 C_t^2 k_{xx}(t,C_t) \Big ] dt + \int _0^\tau \sigma C_t k_x(t,C_t)\,dB_t . \end{aligned}$$

Under the usual integrability assumptions, the stochastic integral is a martingale with zero mean, and applying optional sampling yields

B18 $$\begin{aligned} -k(0,c_0) = \mathbb {E}\left[ \int _0^\tau \Big ( \partial _t k(t,C_t) + (f(t)-\eta C_t) k_x(t,C_t) + \tfrac{1}{2} \sigma ^2 C_t^2 k_{xx}(t,C_t) \Big ) dt\right] . \end{aligned}$$

On the other hand, by the definition of *k*,

$$\begin{aligned} k(0,c_0) = \mathbb {E}\left[ \tau \right] = \mathbb {E}\left[ \int _0^\tau 1 \, dt\right] . \end{aligned}$$

Comparing the two expressions shows that the function *k* must satisfy the backward Kolmogorov equation

B19 $$\begin{aligned} \frac{\partial }{\partial t} k(t,x) + \big (f(t)-\eta x\big ) \frac{\partial }{\partial x}k(t,x) + \frac{1}{2}\sigma ^2 x^2 \frac{{\partial }^{2}}{\partial x^2}k(t,x) = -1, \qquad (t,x)\in [0,\infty )\times (0,1). \end{aligned}$$

The boundary condition at the detection threshold is

B20 $$\begin{aligned} k(t,1)=0 \qquad \text {for all } t\ge 0. \end{aligned}$$

We compile this discussion as Lemma 6 in the main text. We do not impose a boundary condition at $x=0$. Probabilistically, starting from any $x\in (0,1)$, the diffusion $C_t$ remains strictly positive and reaches the level 1 without ever hitting 0. Therefore the boundary point $x=0$ plays no role in the hitting problem and does not carry a prescribed boundary value. Instead, the correct boundary behavior is enforced through the following probabilistic principle: Among all nonnegative functions *u* satisfying the backward equation (B19) and the boundary condition Eq. (B20), the mean hitting time *k* is the minimal one; that is,

$$\begin{aligned} k(t,x) \;\le \; u(t,x) \qquad \text {for all } (t,x)\in [0,\infty )\times (0,1). \end{aligned}$$

This minimality requirement replaces the missing boundary condition at $x=0$ and selects the physically meaningful solution corresponding to the expected time to detection. Finally,

$$\begin{aligned} \mathbb {E}\left[ \tau \ \vert \ C(0) = c_0\right] = k(0,c_0), \end{aligned}$$

where *k* is the minimal nonnegative solution of (B19)–(B20).

This partial differential equation (PDE) is more tractable in the special case wherein $f(t) \equiv c$ for some constant *c*. By removing the time dependency, we drop the PDE into a ODE. To that extent, we consider the situation where the underlying tumor population has reached the equilibrium state. As such, given the stationary state in the ODE, we have *f*(*t*) would not have a time dependency and can be viewed as a constant, say $f^*$. In this sense, we can consider *C*(0) as the mean biomarker proportion as the ODE reaches “close” to the equilibrium state. Eq. (B17) provides one such interpretation. Namely, at *t* large enough for the ODE to reach equilibrium and with $\hat{c}_0$ referring to the proportion of biomarker at the very start of the ODE initiation,

$$\begin{aligned} m(t) = \hat{c}_0 e^{-\eta t} + f^*\int _0^t \,e^{-\eta (t - s) }ds \rightarrow \frac{f^*}{\eta }. \end{aligned}$$

We want to find $\mathbb {E}\left[ \tau \, \vert \, C(0)\right]$ for our process. Given the work earlier in this section, we start off with the time-independent version of Eq. (B18). Namely,

$$\begin{aligned} - k(c_0)&= \mathbb {E}\left[ \int _0^\tau \left( f^* k^\prime (C) - \eta C k^\prime (C) + \frac{1}{2} \, k^{\prime \prime }(C) \sigma ^2 C^2 \right) dt \right] \end{aligned}$$

Thus, suppose that *k* satisfies the ODE given by

B21 $$\begin{aligned} \left\{ \begin{aligned} (f^* - \eta x) k^\prime (x) + \frac{1}{2} \, k^{\prime \prime }(x) \sigma ^2 x^2 = -1 , \\ k(1) = 0, \quad k(x)\ge 0 . \end{aligned} \right. \end{aligned}$$

Among all such solutions, we select the minimal nonnegative one. Then we can use this function and the initial population ratio to obtain the average time for detection. Namely,

$$\begin{aligned} \mathbb {E}\left[ \tau \ \vert \ C(0) = c_0\right] = k(c_0). \end{aligned}$$

Thus, We now solve the differential equation presented above. We can rewrite Eq (B21) as

B22 $$\begin{aligned} \left\{ \begin{aligned} k^{\prime \prime }(x) + p(x) k^\prime (x) = q(x) , \\ k(1) = 0, \quad k(x) \ge 0 , \end{aligned} \right. \end{aligned}$$

where

B23 $$\begin{aligned} p(x) = \frac{2(f^* - \eta x)}{\sigma ^2 x^2}, \qquad q(x) =-\frac{2}{\sigma ^2 x^2}. \end{aligned}$$

**Remark:** The coefficients *p* and *q* in (B23) are singular at $x=0$, but we work exclusively on the open interval (0, 1) and do not impose a boundary condition at 0. Probabilistically, this corresponds to the fact that, starting from $c_0\in (0,1)$, the process $C_t$ remains strictly positive and does not reach 0 in finite time. The condition that *k* be the minimal nonnegative solution on (0, 1) with $k(1)=0$ plays the role of the second boundary condition in selecting the physically relevant solution. We start from the homogeneous form to obtain the general solution.

$$\begin{aligned} k^{\prime \prime } + p(x) k^\prime&= 0,\\ e^{- \int _x^1 p(v) dv} (k^{\prime \prime } + p(x) k^\prime )&= 0,\\ e^{- \int _x^1 p(v) dv} k^{\prime \prime } + p(x) e^{- \int _x^1 p(v) dv} k^\prime&= 0,\\ (e^{-\int _x^1 p(v) dv} k^\prime )^\prime&= 0,\\ k^\prime&= -c_2 e^{\int _x^1 p(v) dv},\\ k(x)&= c_2 \int _x^1 e^{\int _y^1 p(v) dv} dy + c_1 \end{aligned}$$

where we have integrated from some arbitrary initial value $x \in \left( 0,1\right)$ to the final value $C(\tau ) = 1$. Thus, we have the two homogeneous solutions given by

$$\begin{aligned} k_1(x) = 1, \qquad k_2(x) = \int _x^1 e^{\int _y^1 p(v) dv} dy . \end{aligned}$$

We calculate the Wronskian for these two solutions:

$$\begin{aligned} W(x)&= k_1 k_2^\prime - k_1^\prime k_2 \\&= k_2^\prime \\&= - e^{\int _x^1 p(v) dv}\ne 0 \, \forall \, x \in (0,1]. \end{aligned}$$

This means that the two solutions are fundamental solutions for the general case. We now use these general case solutions and method of variation of parameters to obtain the particular solution for the non-homogeneous case. It is well known that the particular solution will have the form

B24 $$\begin{aligned} k_p(x)&= k_1 \int _x^1 \frac{k_2(v) q(v)}{W(v)} dv - k_2 \int _x^1 \frac{k_1(v) q(v)}{W(v)} dv \nonumber \\&= \int _x^1 \frac{k_2(v) q(v)}{W(v)} dv - k_2(x) \int _x^1 \frac{q(v)}{W(v)} dv . \end{aligned}$$

For the first term in the right hand side of the above equation,

B25 $$\begin{aligned} \int _x^1 \frac{k_2(v) q(v)}{W(v)} dv&= \int _x^1 \frac{ \int _v^1 e^{\int _y^1 p(z) dz} dy}{- e^{\int _v^1 p(z) dz}} \; q(v) dv=- \int _x^1 \int _v^1 e^{-\int _v^y p(z) dz} dy \; q(v) dv . \end{aligned}$$

Similarly from (B24),

B26 $$\begin{aligned} -\int _x^1 \frac{k_2(x) q(v)}{W(v)} dv \!=\! -\int _x^1 \frac{\int _x^1 e^{\int _y^1 p(z) dz} }{- e^{\int _v^1 p(z) dz}}dy \; q(v) dv \!=\! \int _x^1 \int _x^1 e^{-\int _v^y p(z) dz} dy \; q(v) dv . \end{aligned}$$

Combining Eqs. (B24),(B25) and, (B26), we get

$$\begin{aligned} k_p(x)&= - \int _x^1\int _v^1 e^{-\int _v^y p(z) dz} dy \; q(v) dv + \int _x^1 \int _x^1 e^{-\int _v^y p(z) dz} dy \; q(v) dv\\&= \int _x^1 \int _x^v e^{-\int _v^y p(z) dz} dy \; q(v) dv. \end{aligned}$$

Thus, the general form for our solution is given by

B27 $$\begin{aligned} k(x)&= c_1 + c_2 \int _x^1 e^{\int _y^1 p(v) dv} dy + \int _x^1 \int _x^v e^{-\int _v^y p(z) dz} dy \; q(v) dv , \end{aligned}$$

where $c_1$ and $c_2$ depend on the boundary conditions. From Eq. (B27) we can see that, $k(1) = 0$ implies that $c_1 = 0$. Thus, we have a one parameter family of solutions which we can then employ the second probabilistic condition that the mean time to escape is the minimal non-negative solution. Namely,

$$\begin{aligned}&c_2 \int _x^1 e^{\int _y^1 p(v) dv} dy + \int _x^1 \int _x^v e^{-\int _v^y p(z) dz} dy \; q(v) dv \ge 0 ,\\&\implies c_2 \ge \frac{- \int _x^1 \int _x^v e^{-\int _v^y p(z) dz} dy \; q(v) dv}{\int _x^1 e^{\int _y^1 p(v) dv} dy. } \end{aligned}$$

Globally, we define

$$\begin{aligned} \hat{c} = \sup _{x \in (0,1)}\left( \frac{- \int _x^1 \int _x^v e^{-\int _v^y p(z) dz} dy \; q(v) dv}{\int _x^1 e^{\int _y^1 p(v) dv} dy } \right) . \end{aligned}$$

Thus, conditional on the fact that $C_0 = c_0 \in (0,1]$, we can then find the mean time for escape to be given by

B28 $$\begin{aligned} \mathbb {E}\left[ \tau \ \vert \ C(0) = c_0\right] = \sup _{x \in (0,1)}&\left( \frac{- \int _x^1 \int _x^v e^{-\int _v^y p(z) dz} dy \; q(v) dv}{\int _x^1 e^{\int _y^1 p(v) dv} dy } \right) \cdot \int _{c_0}^1 e^{\int _y^1 p(v) dv} dy \nonumber \\&+ \int _{c_0}^1 \int _{c_0}^v e^{-\int _v^y p(z) dz} dy \; q(v) dv , \end{aligned}$$

where $p,\,q$ are as defined in Eq (B23). The above discussion for the mean hitting times for the geometric SDE is summarized in Theorem 7 in the main text.

#### B.1.4 Properties of the Solution of the CIR-like SDE

In this section, we consider the SDE given by

B29 $$\begin{aligned} dC =&\left[ f(E,B,I) - \eta C\right] dt + \hat{\sigma } \sqrt{C} dW(t) \nonumber \\ =&\eta \left[ \frac{f}{\eta } - C\right] dt + \hat{\sigma } \sqrt{C} dW(t). \end{aligned}$$

The non-linear formulation for the diffusion term leads to an inability to obtain a closed-form pathwise solution. However, one can obtain the exact transition distribution and generate exact samples for simple functional forms of *f*. In addition, the CIR process is non-negative if it satisfies the Feller condition,

$$2 \eta \alpha = 2 f > \hat{\sigma }^2.$$

This condition cannot be easily guaranteed for time-dependent *f* through the whole trajectory of the stochastic process. As such, neither can non-negativity of the CIR process. As a workaround, we redefine the function *f* by shifting it above this $\hat{\sigma }^2$ value. Namely, consider $\hat{f}(t) = f(t) + \xi$, where $\xi > \frac{ \hat{\sigma }^2}{2}$. Using the SDE given in Eq. (B29) we obtain the first and second moment formulae, which we show below.

#### B.1.5 Proof for Theorem 5

**Mean: **Let $m(t) = \mathbb {E}\left[ C(t)\right]$ then *m*(*t*) satisfies the ordinary differential equation given by

$$ \frac{dm(t)}{dt} = \hat{f}(t) - \eta m(t), \quad m_0 = c_0. $$

This is nothing but the same first order linear ordinary differential equation as in the case of the linear stochastic differential equation case. Thus, the mean of C(t) can be explicitly written as

B30 $$\begin{aligned} m(t) = c_0 e^{-\eta t} + \int _0^t \hat{f}(s) \,e^{-\eta (t - s) }ds. \end{aligned}$$

**Variance: ** Consider $h(x) = x^2$. Using the Itô formulae on *h*(*C*), we have

$$\begin{aligned} dh(C)&= h^\prime (C) dC + \frac{1}{2} h^{\prime \prime }(C) (dC)^2 \\&= \left[ 2 C \hat{f}(t) - 2 \eta C^2 + \hat{\sigma }^2 C \right] dt + 2 \hat{\sigma } C^{\frac{3}{2}} dW(t) . \end{aligned}$$

Taking the expectation of both sides and using the fact that the Itô integral is zero mean, we have that

$$\begin{aligned} \frac{d(\mathbb {E}\left[ C(t)^2\right] )}{dt} = 2 \mathbb {E}\left[ C(t)\right] \hat{f}(t) - 2 \eta \mathbb {E}\left[ C(t)^2\right] + \hat{\sigma }^2 \mathbb {E}\left[ C(t)\right] . \end{aligned}$$

Let $v(t) =\mathbb {E}\left[ C(t)^2\right]$ then the variance of the stochastic differential equation given by Eq (B30) solves the ODE given by

$$\begin{aligned} \frac{dv(t)}{dt} = \left( 2 \hat{f}(t) + \hat{\sigma }^2 \right) m(t)- 2 \eta v(t), \quad v_0 = c_0^2. \end{aligned}$$

Noting that the above equation is a linear first order equation, we can use the integrating factor, $e^{2 \eta t}$. Multiplying both sides of the above equation, we get

$$\begin{aligned} e^{2 \eta t} \frac{dv(t)}{dt} + 2 \eta e^{2 \eta t} v(t)&= \left( 2 \hat{f}(t) + \hat{\sigma }^2 \right) m(t) e^{2 \eta t}, \\ \frac{d}{dt} \left( e^{2 \eta t} v(t) \right)&= \left( 2 \hat{f}(t) + \hat{\sigma }^2 \right) m(t) e^{2 \eta t}. \end{aligned}$$

Thus,

$$\begin{aligned} v(t)&= c^2_0 e^{- 2 \eta t} + \int _0^t \left( 2 \hat{f}(s) + \hat{\sigma }^2 \right) e^{ - 2 \eta (t - s)} m(s) ds \\&= c^2_0 e^{- 2 \eta t} + \int _0^t \left( 2 \hat{f}(s) + \hat{\sigma }^2 \right) \left( c_0 e^{-\eta s} + \int _0^s \hat{f}(u) \,e^{-\eta (s - u) } du\right) ds . \end{aligned}$$

Thus, variance can be found to be

$$\begin{aligned} Var(C(t))&= v(t) - m(t)^2\\&= c^2_0 e^{- 2 \eta t} + \int _0^t \left( 2 \hat{f}(s) + \hat{\sigma }^2 \right) \left( c_0 e^{-\eta s} + \int _0^s \hat{f}(u) \,e^{-\eta (s - u) } du\right) ds - m(t)^2 \\&= c^2_0 e^{- 2 \eta t} + \int _0^t \left( 2 \hat{f} + \hat{\sigma }^2 \right) \left( c_0 e^{-\eta s} + \int _0^s \hat{f}(u) \,e^{-\eta (s - u) } du\right) ds \\&\quad - \left( c_0 e^{-\eta t}+ \int _0^t \hat{f}(s) \,e^{-\eta (t - s) } ds \right) ^2. \end{aligned}$$

The above discussion about variance of the CIR SDE is summarized in Theorem 5 in the main text.

#### B.1.6 Proof for Lemma 6 and Theorem 7 for CIR Case

This underlying mathematics within this section for the CIR SDE given by Eq. (B29) is identical to the work for the mean detection time approach given in Section B.1.3. Here we highlight the key differences. We start from the backward equation for *k* and apply the Itô lemma to the process $k(t,C_t)$. Namely,

$$\begin{aligned} dk(t,C_t)&= \frac{\partial }{\partial t} k(t,C_t)\,dt + \frac{\partial }{\partial x}k(t,C_t)\,dC_t + \frac{1}{2}\frac{{\partial }^{2}}{\partial x^2}k(t,C_t)(dC_t)^2 \\&= \Big [ \frac{\partial }{\partial t} k(t,C_t) + \big (f(t)-\eta C_t\big ) \frac{\partial }{\partial x}k(t,C_t) + \tfrac{1}{2} \hat{\sigma }^2 C_t \frac{{\partial }^{2}}{\partial x^2}k(t,C_t) \Big ]dt\\&\qquad + \hat{\sigma } \sqrt{C_t} \frac{\partial }{\partial x}k(t,C_t)dB_t . \end{aligned}$$

Similarly, in order to find the mean detection time, the function *k* must satisfy the backward Kolmogorov equation

B31 $$\begin{aligned} \frac{\partial }{\partial t} k(t,x) + \big (f(t)-\eta x\big ) \frac{\partial }{\partial x}k(t,x) + \frac{1}{2} \frac{{\partial }^{2}}{\partial x^2}\hat{\sigma }^2 x \frac{{\partial }^{2}}{\partial x^2}k(t,x) = -1, \qquad (t,x)\in [0,\infty )\times (0,1). \end{aligned}$$

The boundary condition at the detection threshold is

B32 $$\begin{aligned} k(t,1)=0 \qquad \text {for all } t\ge 0. \end{aligned}$$

Alongside the same probabilistic condition that among all nonnegative functions *u* satisfying the backward equation (B31) and the boundary condition (B32), the mean hitting time *k* is the minimal one; that is,

$$\begin{aligned} k(t,x) \;\le \; u(t,x) \qquad \text {for all } (t,x)\in [0,\infty )\times (0,1). \end{aligned}$$

Similar to the geometric SDE case, this PDE is more tractable in the special case wherein $f(t) \equiv c$ for some constant *c*. By removing the time dependency, for $x \in (0,1)$, we get

$$\begin{aligned} \big (\hat{f}^*-\eta x\big ) k^{\prime }(x) + \frac{1}{2} \hat{\sigma }^2 x k^{\prime \prime }(x) = -1,\\ \iff k^{\prime \prime }(x) + \hat{p}(x) k^\prime (x) = \hat{q}(x), \end{aligned}$$

where,

B33 $$\begin{aligned} \hat{p} = \frac{2 \big (\hat{f}^*-\eta x\big )}{ \hat{\sigma }^2 x}, \qquad \hat{q} =-\frac{2}{ \hat{\sigma }^2 x}. \end{aligned}$$

Note that this is the same ODE as in Eq. (B22). As such, we can follow the same work to obtain the minimal non-negative solution. As such, we have

B34 $$\begin{aligned} \mathbb {E}\left[ \tau \ \vert \ C(0) = c_0\right] = \sup _{x \in (0,1)}&\left( \frac{- \int _x^1 \int _x^v e^{-\int _v^y \hat{p}(z) dz} dy \; \hat{q}(v) dv}{\int _x^1 e^{\int _y^1 \hat{p}(v) dv} dy } \right) \cdot \int _{c_0}^1 e^{\int _y^1 \hat{p}(v) dv} dy \nonumber \\&+ \int _{c_0}^1 \int _{c_0}^v e^{-\int _v^y \hat{p}(z) dz} dy \; \hat{q}(v) dv , \end{aligned}$$

where $\hat{p},\,\hat{q}$ are as defined in Eq (B33). The above discussion for the mean hitting times for the CIR SDE is can be treated as a restatement of Lemma 6 and Theorem 7 in the main text.

## Declarations

### Code availability

The code used to generate the figures are available on https://github.com/TAMUGeorgeGroup/ODE_SDE_Model.
