# Supplementary material for: Coupled SDE-ODE Modeling of Tumor-Immune Dynamics to Infer Biomarker Release
Source: Bull Math Biol. 2026 Jun 23;88(7):121. doi: 10.1007/s11538-026-01665-9 (PMC13290969; doi:10.1007/s11538-026-01665-9)
Supplement: Supplementary file 1 — (pdf 8646 KB) [file 11538_2026_1665_MOESM1_ESM.pdf]

Supplementary Information: Coupled SDE-ODE Modeling of Tumor-Immune Dynamics to Infer Biomarker Release

Pujan Shrestha, Yijia Fan, and Jason T. George

March 30, 2026

S1 Nondimensionalization of the ODE Model

For ease of fitting to data, we also consider the nondimentionalized version of the model. Nondimensionalization of an ODE model helps reduce the number of parameters by combining them into dimensionless groups, thus simplifying the system. By eliminating units and resealing variables, the model becomes easier to analyze, as fewer distinct parameters need to be considered. This reduction is particularly useful for our case. We first scale the tumor populations by their carrying capacity. Furthermore, we assume that there is a reference I_0 that we can obtain empirically or as a parameter from the agent based model. Then,

$$\bar{E} = \frac{E}{K} \qquad \bar{B} = \frac{B}{K} \qquad \bar{I} = \frac{I}{I_0}. \tag{S1}$$

Now we scale time by the growth rate of the baseline recognizable tumor subpopulation. Namely, let $\tau = \gamma t$. Under these scaling, we have the scaled evasive tumor dynamics,

$$\frac{d\bar{E}}{d\tau} = \frac{1}{\gamma K}\frac{dE}{dt} = \frac{r}{\gamma}\bar{E}\left(1 - \bar{E} - p\bar{B}\right) - \frac{\alpha I_0}{\gamma}\bar{E}\bar{I}.$$

For the baseline recognizable tumor subpopulation,

$$\frac{d\bar{B}}{d\tau} = \frac{1}{\gamma K}\frac{dB}{dt} = \bar{B}(1 - q\bar{E} - \bar{B}) - \frac{\beta I_0}{\gamma}\bar{B}\bar{I}.$$

Finally, the immune compartment becomes

$$\frac{d\bar{B}}{d\tau} = \frac{1}{\gamma I_0}\left(aEI + bBI - \delta I\right) = \frac{aK}{\gamma}\bar{E}\bar{I} + \frac{bK}{\gamma}\bar{B}\bar{I} - \frac{\delta}{\gamma}\bar{I}.$$

Thus, we have a new system of equations,

$$\begin{aligned} d\bar{E} &= \bar{r}\bar{E}\left(1 - \bar{E} - p\bar{B}\right) - \bar{\alpha}\bar{E}\bar{I}, \\ d\bar{B} &= \bar{B}(1 - q\bar{E} - \bar{B}) - \bar{\beta}\bar{B}\bar{I}, \\ d\bar{I} &= \bar{a}\bar{E}\bar{I} + \bar{b}\bar{B}\bar{I} - \bar{\delta}\bar{I}, \end{aligned} \tag{S2}$$

where the re-scaled parameters are

$$\bar{r} = \frac{r}{\gamma}, \qquad \bar{\alpha} = \frac{\alpha I_0}{\gamma}, \qquad \bar{\beta} = \frac{\beta I_0}{\gamma}, \qquad \bar{a} = \frac{aK}{\gamma}, \qquad \bar{b} = \frac{bK}{\gamma}, \qquad \bar{\delta} = \frac{\delta}{\gamma}.$$

S2 Figures and Results for Biomarker Profile ($\sigma^2 < 2\eta$)

- **Baseline tumor eliminated with evasive-immune Coexistence:**

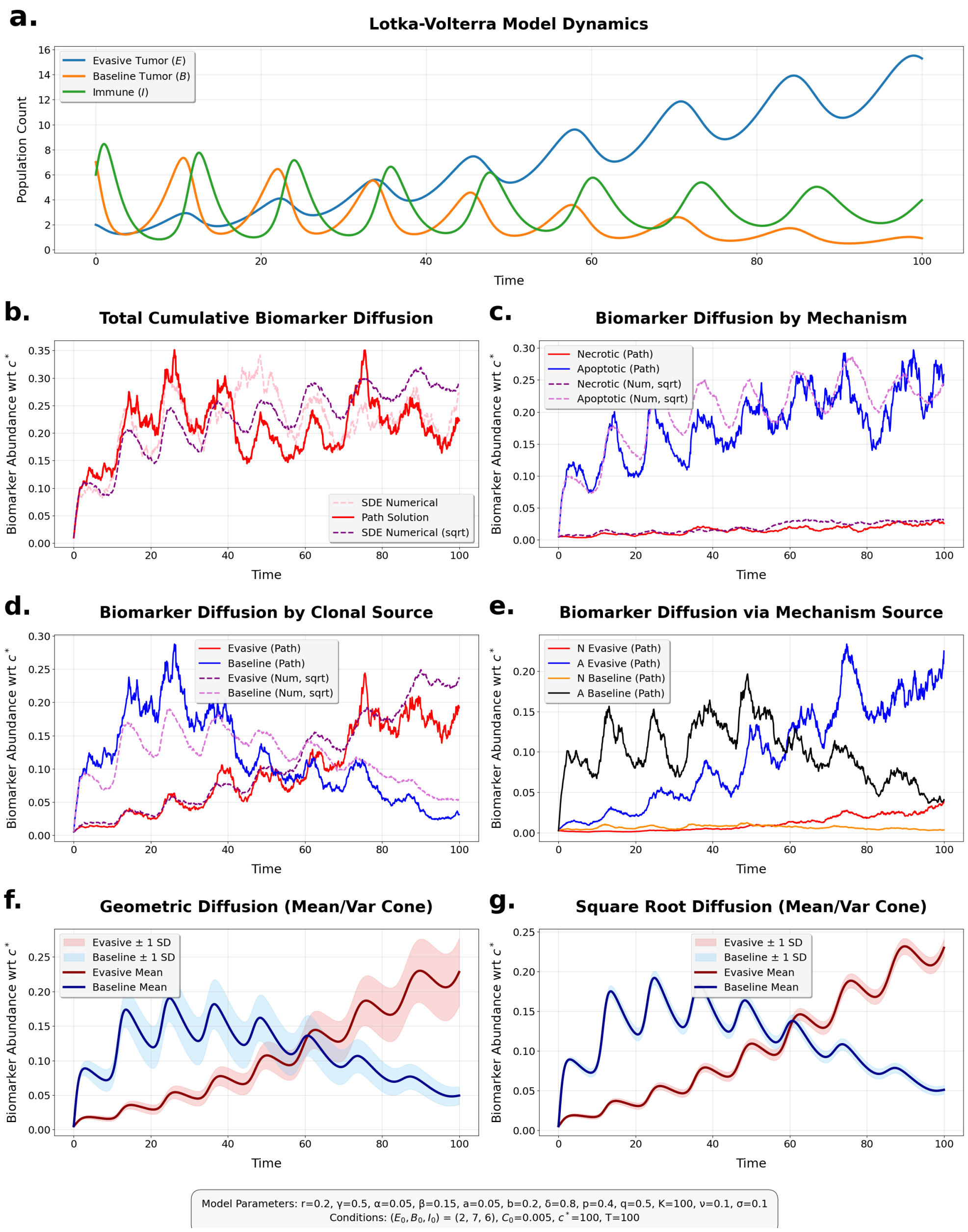

Figure S1: Dynamics of the case where baseline tumor is eliminated with evasive-immune coexistence and associated biomarker release trajectories a) Lotka-Volterra Trajectory b) Cumulative biomarker trajectory c) Necrotic vs Apoptotic Split d) Evasive vs Baseline Split e) Full table split f) Mean and Variance of the biomarker trajectories with geometric diffusion. g) Mean and Variance of the biomarker trajectories with square root diffusion. Dotted lines represent numerical simulations via Euler-Maruyama, lines represent path simulations via Monte Carlo simulations. In all cases, parameter choices provided at the bottom of the figure.

- **Heterogeneous tumor coexistence:**

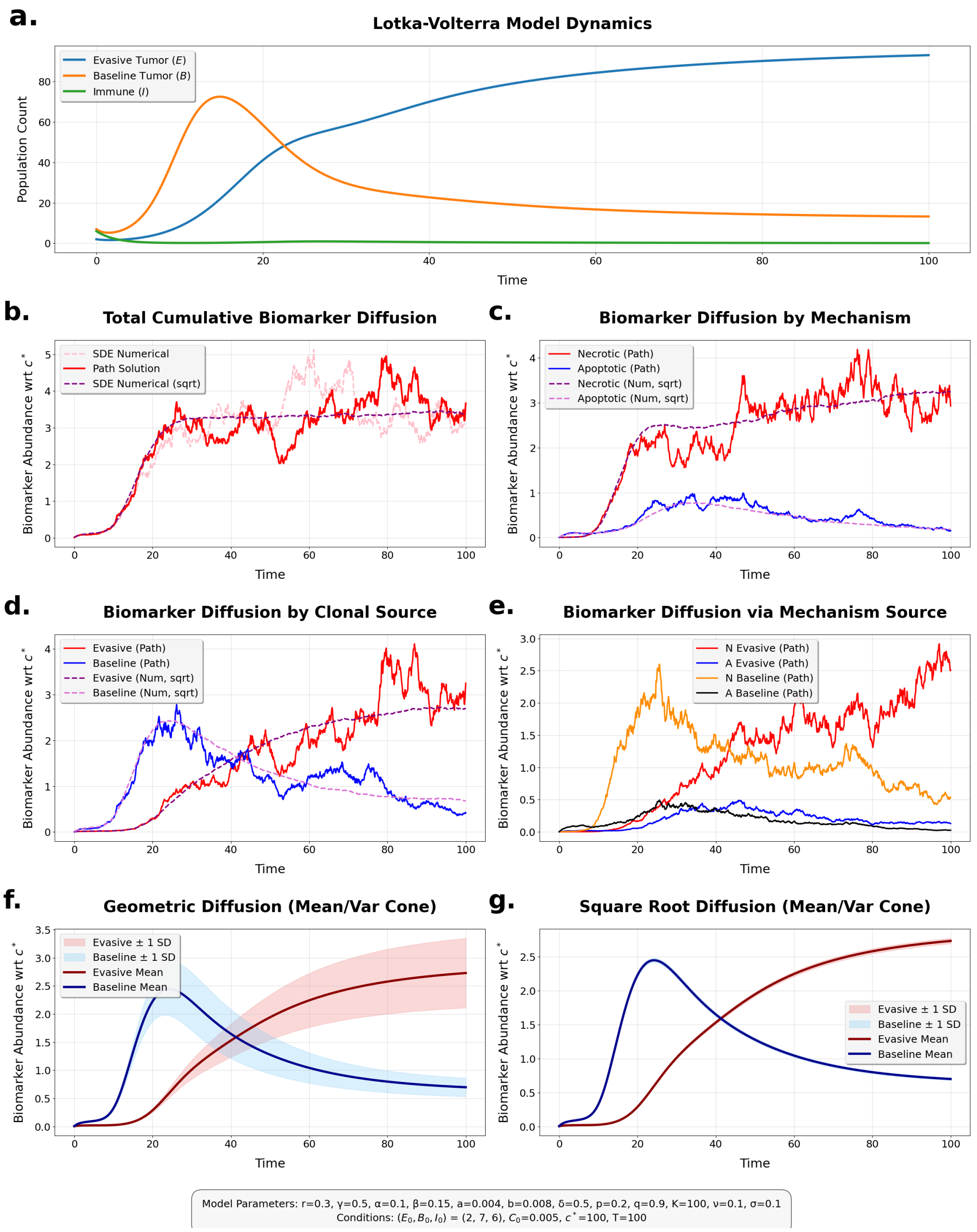

Figure S2: Dynamics of the case with heterogeneous tumor coexistence with the immune compartment eliminated and associated biomarker release trajectories a) Lotka-Volterra Trajectory b) Cumulative biomarker trajectory c) Necrotic vs Apoptotic Split d) Evasive vs Baseline Split e) Full table split f) Mean and Variance of the biomarker trajectories with geometric diffusion. g) Mean and Variance of the biomarker trajectories with square root diffusion. Dotted lines represent numerical simulations via Euler-Maruyama, lines represent path simulations via Monte Carlo simulations. In all cases, parameter choices provided at the bottom of the figure.

- **Evasive tumor prevails:**

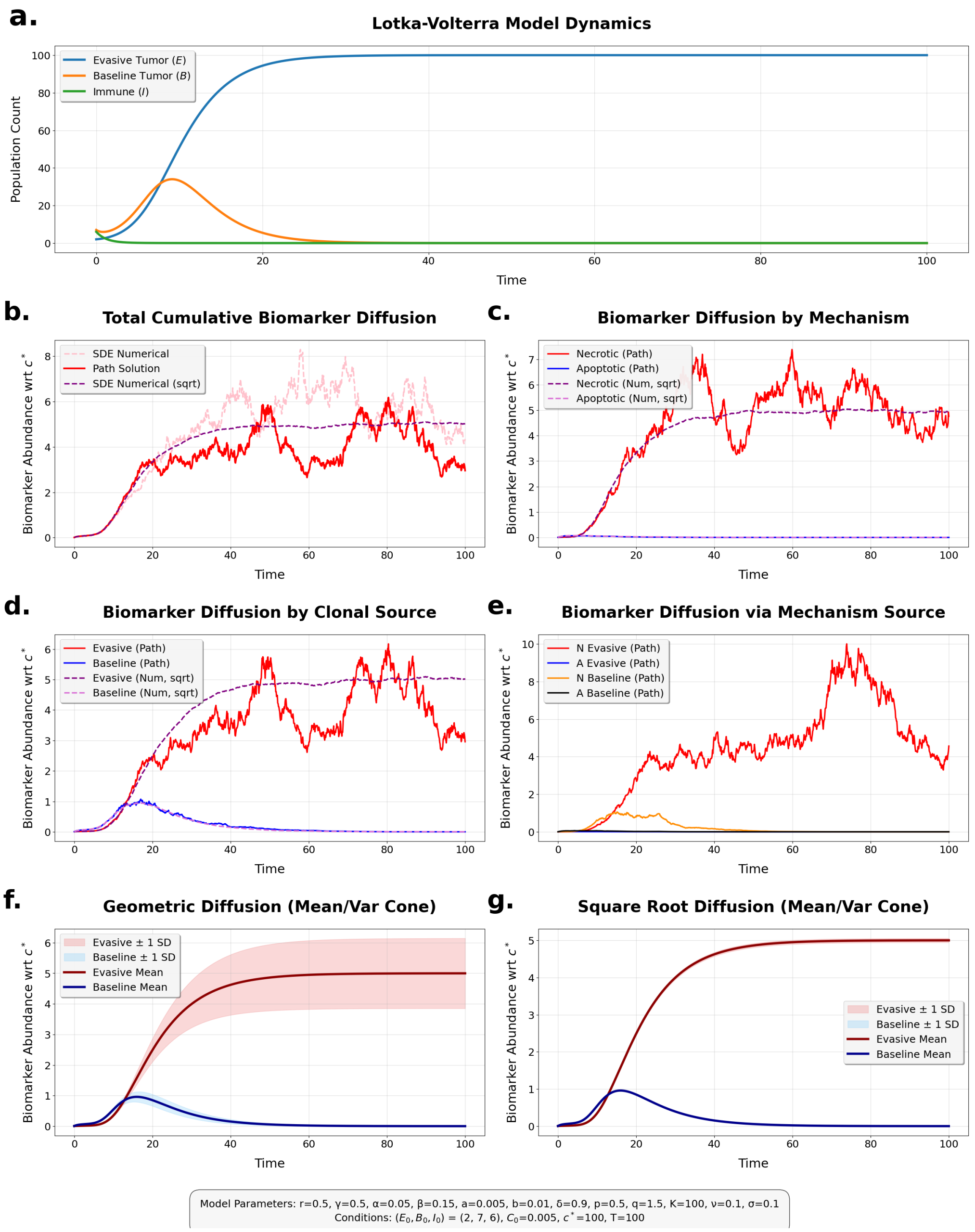

Figure S3: Dynamics of the case with evasive tumor subpopulation prevails and associated biomarker release trajectories a) Lotka-Volterra Trajectory b) Cumulative biomarker trajectory c) Necrotic vs Apoptotic Split d) Evasive vs Baseline Split e) Full table split f) Mean and Variance of the biomarker trajectories with geometric diffusion. g) Mean and Variance of the biomarker trajectories with square root diffusion. Dotted lines represent numerical simulations via Euler-Maruyama, lines represent path simulations via Monte Carlo simulations. In all cases, parameter choices provided at the bottom of the figure.

S3 Parameter Sweeps for the Hitting Time for Interior Equilibrium Case

In what follows, we parametrize the underlying LV model for the heterogeneous tumor-immune coexistence state. We then perform parameter sweeps to understand how the mean hitting time is impacted by the parameters in the LV system.

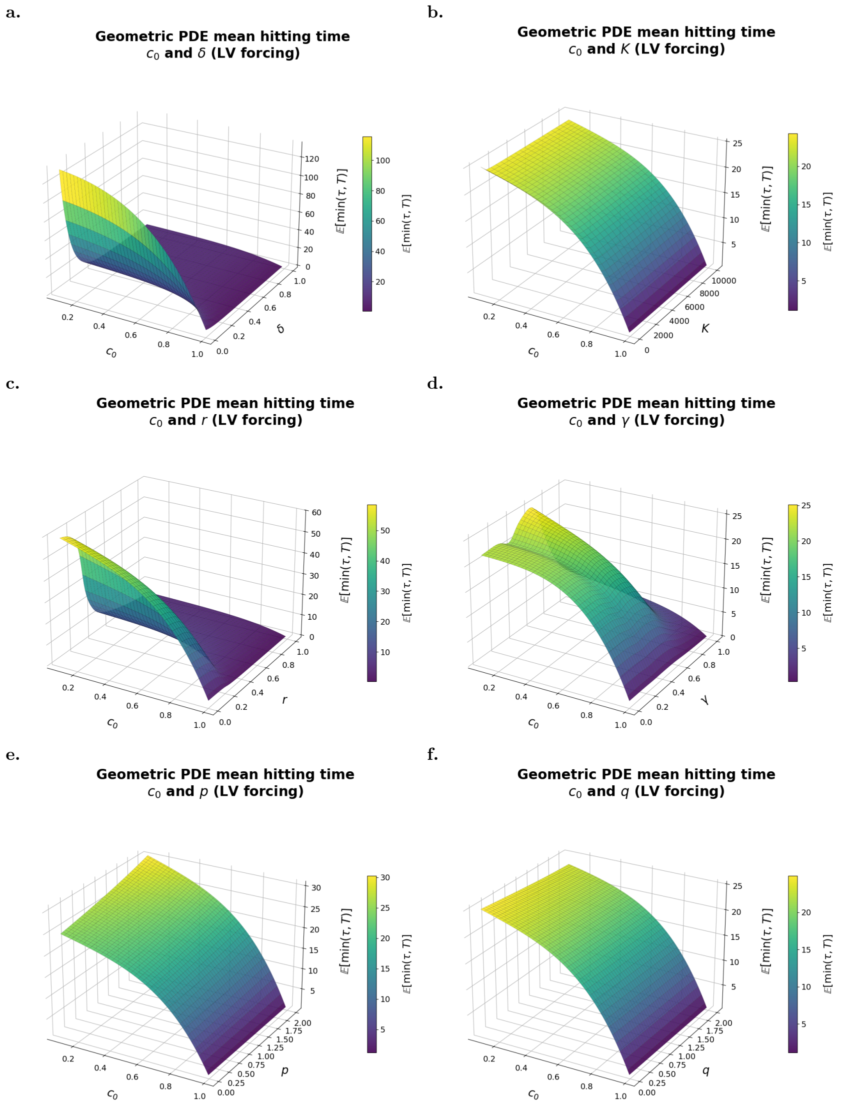

Figure S4: Impact of underlying LV model to the expected hitting time for the geometric SDE. Parameter sweep using the parametrization for the interior equilibrium state. a) Death rate for the immune compartment b) Carrying capacity c,d) Growth rates for the evasive and baseline tumor compartment e,f) Competition Parameters. Default parameters used: $r = 0.28$, $\gamma = 0.5$, $\alpha = 0.1$, $\beta = 0.2$, $a = 0.00195$, $b = 0.04$, $\delta = 0.2$, $p = 0.1$, $q = 0.09$, $K = 100$, $c^* = 50$, $\sigma = 0.25$, $\eta = 0.1$

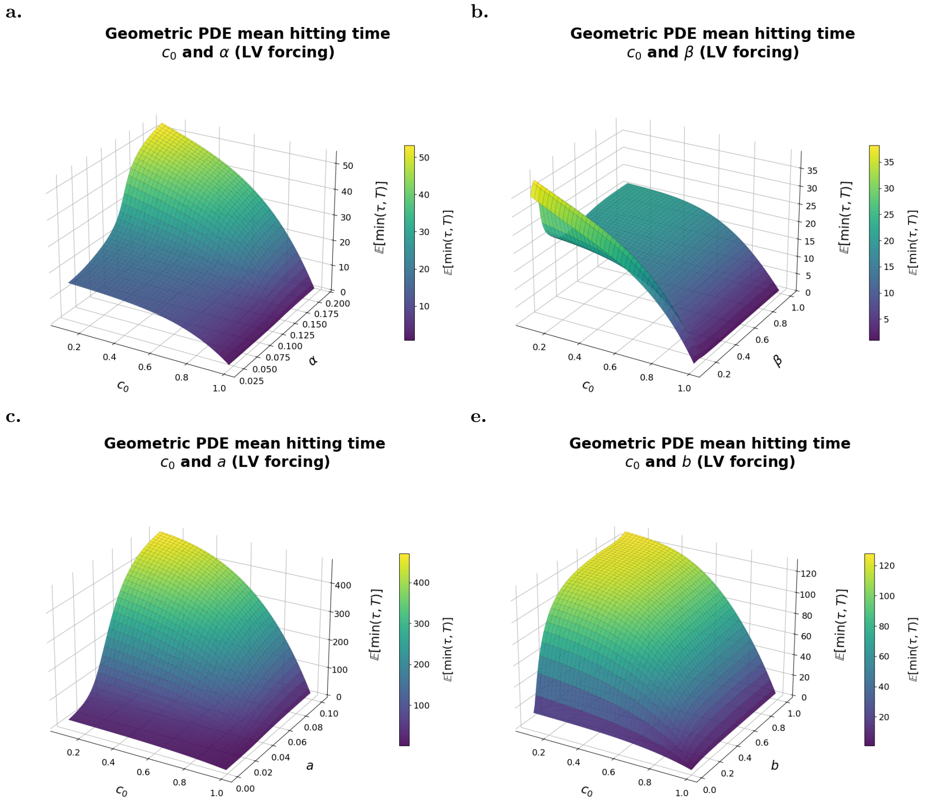

Figure S5: Impact of underlying LV model to the expected hitting time for the geometric SDE. Parameter sweep using the parametrization for the interior equilibrium state. a,b) Death rate for the evasive and baseline compartment c,d) Immune expansion rates for the evasive and baseline tumor compartment. Default parameters used: $r = 0.28$, $\gamma = 0.5$, $\alpha = 0.1$, $\beta = 0.2$, $a = 0.00195$, $b = 0.04$, $\delta = 0.2$, $p = 0.1$, $q = 0.09$, $K = 100$, $c^* = 50$, $\sigma = 0.25$, $\eta = 0.1$

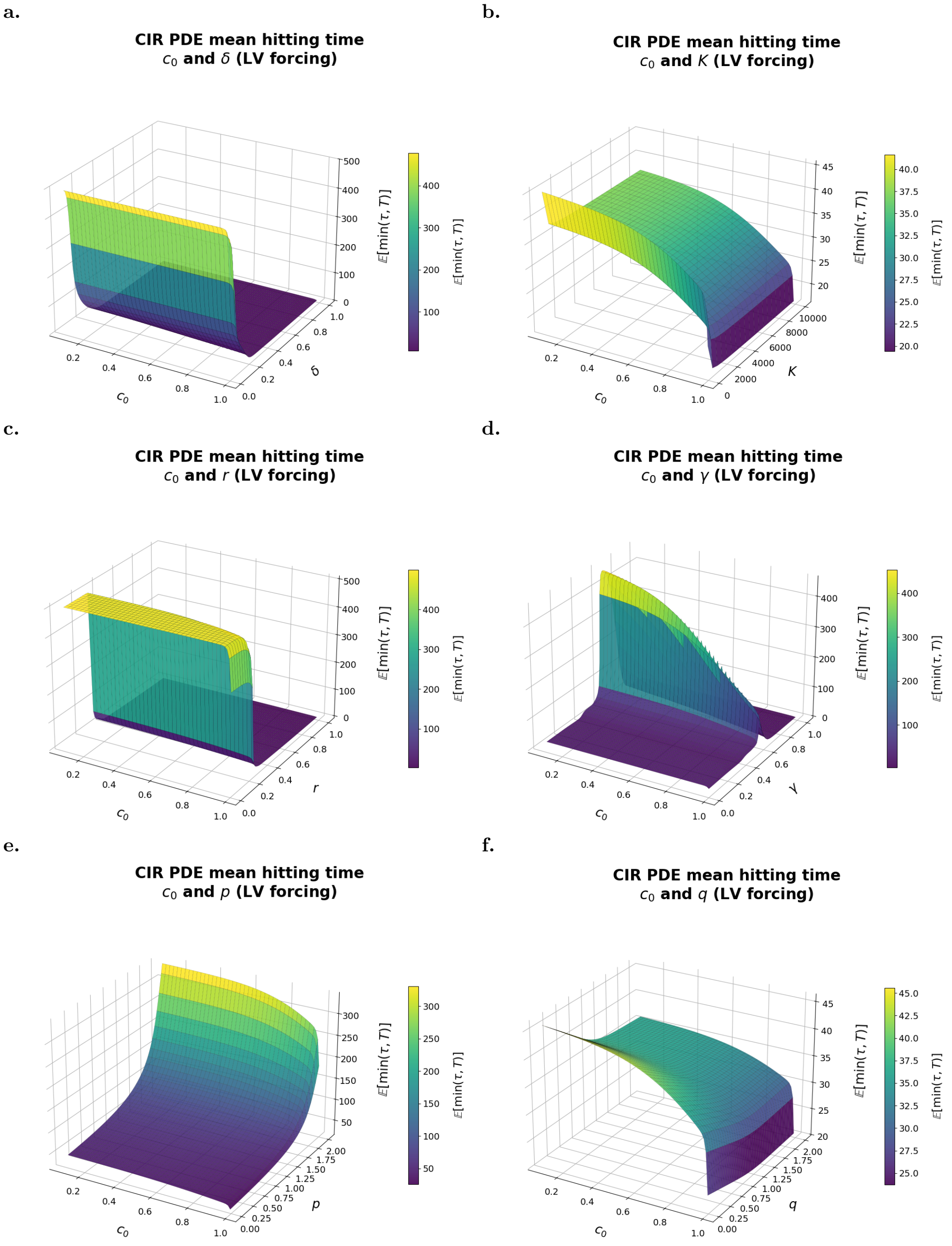

Figure S6: Impact of underlying LV model to the expected hitting time for the CIR SDE. Parameter sweep using the parametrization for the interior equilibrium state. a) Death rate for the immune compartment b) Carrying capacity c,d) Growth rates for the evasive and baseline tumor compartment e,f) Competition Parameters. Default parameters used: $r = 0.28$, $\gamma = 0.5$, $\alpha = 0.1$, $\beta = 0.2$, $a = 0.00195$, $b = 0.04$, $\delta = 0.2$, $p = 0.1$, $q = 0.09$, $K = 100$, $c^* = 50$, $\sigma = 0.25$, $\eta = 0.1$

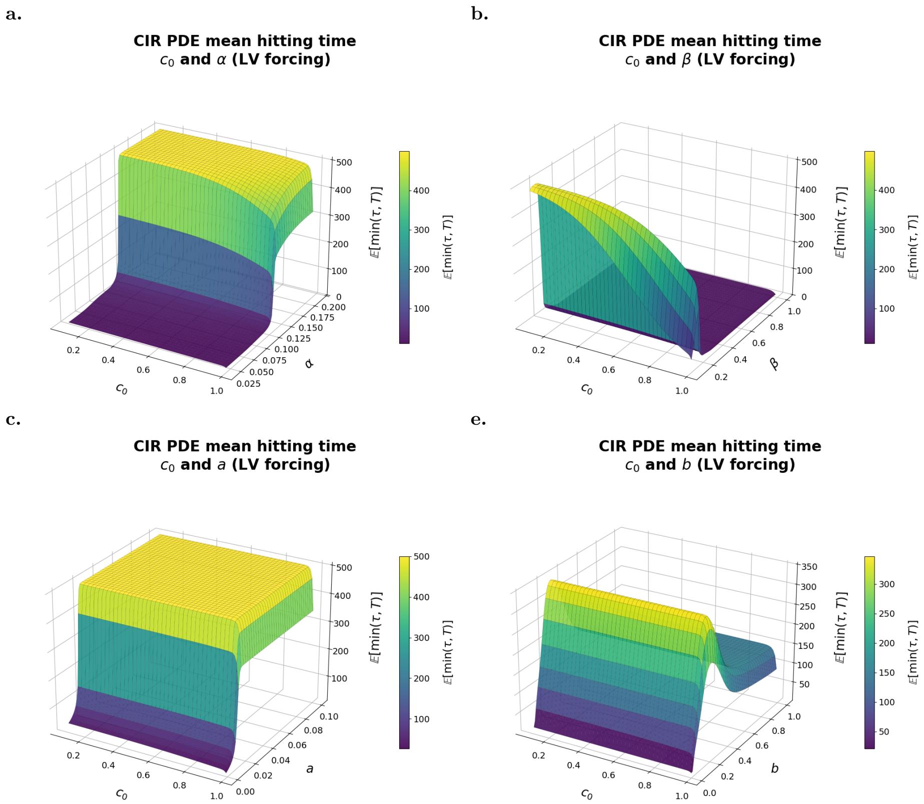

Figure S7: Impact of underlying LV model to the expected hitting time for the CIR SDE. Parameter sweep using the parametrization for the interior equilibrium state. a,b) Death rate for the evasive and baseline compartment c,d) Immune expansion rates for the evasive and baseline tumor compartment. Default parameters used: $r = 0.28$, $\gamma = 0.5$, $\alpha = 0.1$, $\beta = 0.2$, $a = 0.00195$, $b = 0.04$, $\delta = 0.2$, $p = 0.1$, $q = 0.09$, $K = 100$, $c^* = 50$, $\sigma = 0.25$, $\eta = 0.1$
